# Polymer/Carbon-Based Hybrid Aerogels: Preparation, Properties and Applications

**DOI:** 10.3390/ma8105343

**Published:** 2015-10-09

**Authors:** Lizeng Zuo, Youfang Zhang, Longsheng Zhang, Yue-E Miao, Wei Fan, Tianxi Liu

**Affiliations:** 1 State Key Laboratory of Molecular Engineering of Polymers, Department of Macromolecular Science, Fudan University, 220 Handan Road, Shanghai 200433, China; 14110440025@fudan.edu.cn (L.Z.); 13110440035@fudan.edu.cn (Y.Z.); 13210440035@fudan.edu.cn (L.Z.); 2 State Key Laboratory of Modification of Chemical Fibers and Polymer Materials, College of Materials Science and Engineering, Donghua University, 2999 North Renmin Road, Shanghai 201620, China; 12110440023@fudan.edu.cn

**Keywords:** polymer aerogels, carbon aerogels, hybrids, porous, three-dimensional network

## Abstract

Aerogels are synthetic porous materials derived from sol-gel materials in which the liquid component has been replaced with gas to leave intact solid nanostructures without pore collapse. Recently, aerogels based on natural or synthetic polymers, called polymer or organic aerogels, have been widely explored due to their porous structures and unique properties, such as high specific surface area, low density, low thermal conductivity and dielectric constant. This paper gives a comprehensive review about the most recent progresses in preparation, structures and properties of polymer and their derived carbon-based aerogels, as well as their potential applications in various fields including energy storage, adsorption, thermal insulation and flame retardancy. To facilitate further research and development, the technical challenges are discussed, and several future research directions are also suggested in this review.

## 1. Introduction

The term aerogel is to designate a sol-gel material that the liquid component of the gel has been replaced with gas to leave an intact solid nanostructure without pore collapse in which 90%~99% is air by volume [1,2,3]. The density of aerogel ranges from 1000 kg·m^−3^ (above the solid density) to 1 kg·m^−3^ (lower than the density of air), which induces dramatic changes in the properties [4]. Due to the high porosity as well as dual microscopic (*i.e.*, nanoscale skeleton) and macroscopic (*i.e.*, condensed state matter) structural features, aerogel exhibits versatile unique properties such as ultralow thermal conductivity, sonic velocity and dielectric constant, high specific surface area, as well as ultrawide adjustable density and refractive index [5,6,7,8,9,10]. Therefore, it is an attractive material for applications in chemical sensors, thermal insulations, chemical adsorbents, catalysts or catalytic carriers, and space explorations.

**Figure 1:**
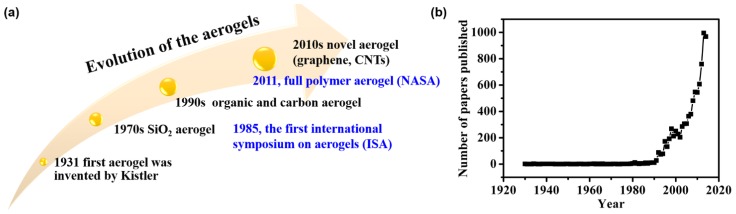
(**a**) Evolution of the aerogels. (**b**) The number of papers published every year during the last decades (search by aerogel from Web of Science).

Evolution of the aerogels is illustrated in Figure 1a, and the number of papers published on aerogels is booming, especially in the last 20 years (Figure 1b). Aerogel was first invented by Kistler in 1931 [11], which was named as “aerogel” (air + gel) because the liquid component inside the wet gel was replaced by air without damaging the solid microstructure. This silica aerogel was fabricated using sodium silicate (that is water-glass) as the precursor of silica, exhibiting some fantastic properties. Nevertheless, aerogel did not arouse broad interests due to the high manufacturing cost, tedious and time-consuming procedures during the synthesis process. The significant innovation was the replacement of water-glass/water systems with organic precursors and the corresponding organic solvents to facilely prepare aerogels, which was represented by the usage of tetramethyorthosilicate by Teichner’s group in 1968 [12] and safer tetraethylorthosilicate by Russo *et al.*, in 1986 [13], and the development of carbon dioxide (CO_2_) supercritical fluid drying [14]. However, aerogel research was limited to silica and several inorganic compositions only during about 70 years after the aerogel was invented. However, at the end of the 1980s, a new type of aerogel was invented by Pekala through the usage of an organic polymer, resorcinol-formaldehyde (RF), during the sol-gel process to develop organic and carbon aerogels [15]. Indeed, it was the right beginning of organic or polymer aerogels. Afterwards, the chemical composition of organic aerogels has been progressively diversified and the family of organic aerogels steadily increases. Various organic aerogels have been prepared, including polyimide (PI)-based aerogels [16], poly(vinyl alcohol) (PVA)-based aerogels [17] and poly(vinyl chloride)-based aerogels [18]. Additionally, aerogels derived from supramolecular gels, which are formed by the self-assembly of low molecular weight organic gelators, pave the way for their developments in high-tech materials and biomaterials [19,20,21]. At the same time, it is found that the polymer that makes up an organic aerogel can be dehydrated after heating to certain temperatures in an inert atmosphere (such as nitrogen or argon), leaving behind the carbon aerogel. Unlike silica aerogel, the carbon aerogel is an electrical conductor, which is called the aero-capacitor and characterized as an “electrochemical double capacitor with high power density and high energy density” [22]. After entering the 21st century, research on aerogels was booming with new types of aerogels emerging, such as non-silica aerogels [23,24,25,26], chalcogenide aerogels [27,28,29,30,31], gradient aerogels and other aerogel composites [32,33,34,35,36,37,38,39,40]. More recently, novel aerogels such as carbon nanotube (CNT) aerogels [41,42,43,44,45,46], graphene aerogels [47,48,49,50,51], silicon aerogels and carbide or carbonitride aerogels [52,53,54,55] were continually added into the aerogel community.

Besides single-component aerogels, aerogels with multi-components were also fabricated to meet the ever-increasing desire for high performance and multi-functions. Indium-tin oxide aerogel, yttria-stabilized zirconia aerogel and binary oxide aerogel have been designed as oxide conductors or catalysts [56,57,58]. For example, ultralight metal oxide/silica aerogels combine the function of metal oxide and the rigid microstructure of the silica aerogel, which can greatly increase the efficiency for laser-X-ray conversion [59]. Moreover, organic aerogels containing various inorganic fillers such as clay, metal oxides or CNT/graphene were synthesized with improved performance and expanded applications in various fields [17,32,33,34,35,36,37,39]. In addition, the preparation of metal oxide/carbon composite aerogels is presumably a good way to improve the capacitive properties of carbon aerogels considerably [60,61,62].

Even though silica aerogels are the most widely studied, potential applications of silica aerogels in aerospace, industry, and daily life have been restricted because of their poor mechanical properties and hygroscopic nature. On the other hand, polymer aerogels including cellulose-based, resin-based, PI-based and their derived carbon aerogels exhibit diverse structures and properties, which widely broaden the application of aerogel based materials. Furthermore, hybrid aerogels with multi-components show enhanced performance and multi-functions, which are attracting more and more interest recently. Therefore, in this paper, recent advances in the field of polymer/carbon aerogels and their derivative hybrid aerogels will be discussed, with particular emphasis placed on their fabrication/synthesis methods, structures, properties and applications.

## 2. Preparation of Polymer/Carbon Aerogels

**Figure 2:**
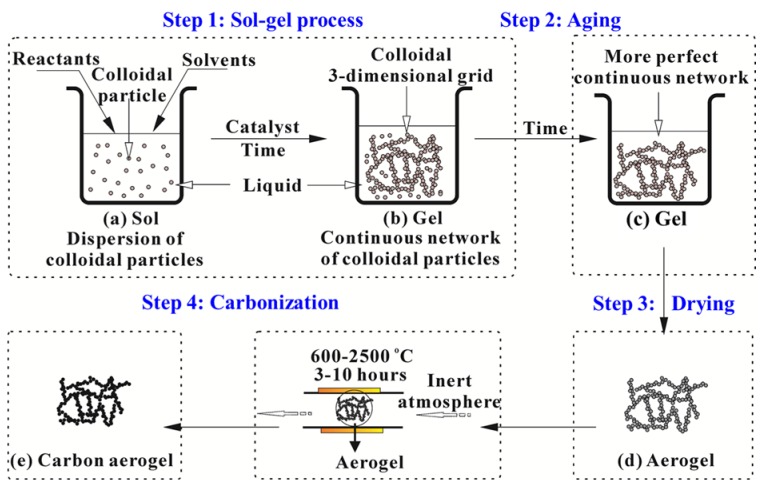
General preparation process of polymer or carbon aerogels.

The potential application of the aerogel is based on its properties, which is strongly relied on the microstructure. Therefore, it is very important to realize the microstructure control during the preparation process. Normally, the preparation process of the aerogel includes following three or four steps (Figure 2):

(i) Sol-gel transition (gelation): Nanoscale sol particles are crosslinked and hierarchically assembled into a wet gel spontaneously or catalyzed by catalysts via hydrolysis or condensation reactions.
(ii) Network perfection (aging): Mechanically reinforce the tenuous solid skeleton generated during the sol-gel process.
(iii) Gel-aerogel transition (drying): The solvent inside the wet gel is replaced by air without serious microstructure damage.

All three steps could determine the microstructure of the aerogels and affect their properties and applications. Besides, it is worth to mention that an additional carbonization process (step 4, Figure 2) is required for fabricating carbon aerogels. In the following sections, the basic preparation procedure of aerogels including the sol-gel process, aging, drying methods and carbonization will be introduced in details.

### 2.1. The Sol-Gel Process

The sol-gel process designates the transition of a colloidal solution into a disordered, branched and continuous network linked by colloidal particles, which is interpenetrated by a liquid at low temperature (typically *T* < 100 °C). This gelation process can take place by the aggregation of colloidal particles via destabilizing the sol, or by hydrolysis and polycondensation of organometallic precursors, or by polycondensation of water-glass. The second route is used most frequently for the preparation of aerogels, which allows the most convenient variation of reaction parameters. Using this route, aerogels of main group oxides, transition and semimetal oxides, as well as organic compounds, can be produced.

Typically, there are two steps in the sol-gel process [63,64]. The first step is the dissolution of monomers in a solvent. Then, independent solid colloidal particles with the size of approximately 2–10 nm are formed as a result of the polymerization process, which involves the establishment of chemical bonds between the monomers. Thus, a colloidal suspension also termed a sol is obtained with the solid colloidal particles stably dispersed in the solvent. Sol can be prepared by two techniques, condensation and dispersion of particles. Condensation proceeds by nucleation growth of particles to the appropriate size, whereas dispersion involves the reduction of large particles down to the colloidal dimensions. The size and properties of the resulting particles depend on the relative rates of these two processes. Sol formation is favored when the rate of nucleation is high and the rate of crystal growth is low. Depending on the degree of crosslinking and the growth process by which they are formed, the inorganic clusters can be either colloidal or polymeric in nature and can range from 10 to 200 Å in diameter. Several factors, such as polarity of the solvent, ionic strength of the reaction medium, and temperature, can be used to manipulate the formation of the sol.

In the second step, these colloidal particles are made to link with each other as polymerization process continues or crosslinked by additional crosslinkers in the solvent, so as to build a three-dimensional (3D) open grid which is termed a gel. Sol particles and small clusters in the solution attach to the spanning cluster and extend the gel network as time goes on. Commonly, the gels transformed from a sol by the gelation process are termed colloidal gels. For preparation of aerogels, the gelation is most conveniently induced through a change in the pH of the reaction solution. The mechanical state of the gel depends very much upon the number of cross-links in the network. It is obvious that the greater the degree of cross-linking, the more rigid the structure formed. On the other hand, it is also possible to form some gels directly from linear polymers instead of a precursor solution, without the intermediate occurrence of individual particles. In this condition, the gels are termed polymeric gels.

It is obvious that the sol-gel process allows us to tailor nanostructured materials by choosing appropriate reaction parameters. In addition, the sol-gel process provides the possibility of doping gels with molecular compounds. This can be performed either chemically by using suitable alkoxide derivates, or physically by introducing additives in the porous network. For example, the integration of hydrophobic alkyl groups can improve the water resistance of aerogels and the inclusion of soot can enlarge the infrared extinction.

### 2.2. Aging

When a gel is maintained in its liquid form, its structure and properties continue to change long after the gel point. This process is termed aging. Generally, the aim of such aging step is to mechanically reinforce the tenuous solid skeleton generated during the sol-gel process [65,66].

During aging, four processes can take place singly or simultaneously, including polycondensation, syneresis, coarsening, and phase transformation. Polycondensation reactions continue to occur within the gel network as long as the neighboring sol particles are close enough to react, which effectively increases the connectivity of the network and its fractal dimension. Syneresis is the spontaneous shrinkage of the gel, resulting from the expulsion of liquid from the pores. This process is generally attributed to the formation of new bonds during polycondensation reactions, which increases the bridging bonds and causes contraction of the gel network. As a result, larger particles grow at the expense of smaller ones and necks between the particles are filled up, thus leading to a narrow particle size distribution with larger mean particle diameter and decreased specific surface area but without shrinkage. This phenomenon can be further enhanced by increasing pH, temperature or pressure, resulting in coarsening of the gel structure.

### 2.3. Gel-Aerogel Transition (Drying)

For aerogels, the drying procedure is a very important step in order to preserve the highly porous structure. The gel, still embedded in the solvent, which consists mainly of alcohol with some water and catalyst, is called an alcogel or hydrogel. To remove the liquid, several extraction methods, such as supercritical drying (using CO_2_, acetone, or ethanol) [15,23,25,67,68,69,70,71,72,73,74], ambient pressure drying [24,75,76,77,78,79], freeze-drying [17,35,37,38], microwave drying [80] and vacuum drying [81,82], can be employed. Normal drying at ambient conditions leads to capillary tensions as the vapor/liquid interface retreats into the porous structure. The resulting shrinkage or even cracking of the gel network proceeds until the densified structure is able to withstand these forces. Depending on the aging, the structure of the dry aerogels consists of a more or less densely packed arrangement of gel particles or network chains with the remaining porosity amounts to about 50%. Shrinkage can be reduced by prolonged aging, by aging with additional catalyst, or by using chemical additives.

#### 2.3.1. Supercritical Drying

Supercritical drying is a traditional drying technique that has been extensively studied. This type of drying prevents the formation of a liquid-vapor meniscus that recedes during the emptying of the pores in the wet gels. Practically, supercritical drying consists of heating the wet gel in a closed container, so that the pressure and temperature exceeds the critical temperature, *T_c_*, and critical pressure, *P_c_*, of the solvent entrapped in the pores inside the gel. As in a supercritical fluid, liquid and vapor phase become indistinguishable, and no capillary forces occur. After releasing the fluid through the outlet valve and subsequent cooling, the aerogel can be taken out from the autoclave. It is important that enough solvent is provided in order to guarantee the supercritical conditions throughout the whole drying process, otherwise shrinking and cracking will occur.

Critical conditions are very different depending on the fluid which impregnates the wet gel, and the most commonly used is CO_2_ [83,84]. For example, PI aerogels with an intrinsically hydrophobic nature and a dielectric constant as low as 1.19 were prepared by a supercritical CO_2_ drying procedure [85]. Kobayashi *et al.* [86] reported a structurally new type of aerogel with a 3D ordered nanofiber skeleton of liquid-crystalline nanocellulose by supercritical CO_2_ drying. Furthermore, monolithic pectin aerogels were prepared with the thermal conductivity ranging from 0.016 to 0.022 W·m^−1^·K^−1^ via dissolution gelation coagulation and subsequent drying with supercritical CO_2_ [87].

Compared to other drying methods, supercritical drying is a more effective method for preventing the shrinkage or collapse of mesopores. However, one main disadvantage for supercritical CO_2_ drying is that it is a time-consuming procedure. Therefore, a less time-consuming drying method without solvent exchange, *i.e.*, supercritical acetone drying, has been reported for the good dissolvability of acetone in supercritical CO_2_ [69,71]. The results show that RF aerogels prepared by these two methods have similar chemical composition and microstructures, but the aerogels dried by supercritical acetone show larger shrinkage and pore size. On the other hand, aerogels dried by supercritical ethanol show similar framework with little shrinkage compared to supercritical CO_2_ or acetone drying [68].

#### 2.3.2. Ambient Pressure Drying

Ambient pressure drying is known as a promising technique that can be applied on large scale for industrial purposes. This method relies on a passivation of the pore surface inside the gel, so as to impede further formation of new chemical bonds after condensation reactions when the gel network is compressed under the drying stresses. At the end of the solvent evaporation process, an aerogel monolith is no longer submitted to capillary stresses so that it can resume its wet size by a spring-back effect. For example, Schwan *et al.* prepared RF organic aerogels via a sol-gel process in sodium carbonate solution, followed by solvent exchange with acetone and then dried under ambient pressure condition. Carbon aerogels using watermelon as the starting materials were also prepared through a hydrothermal method, followed by ambient pressure drying. Though ambient pressure drying is a simple and energy-saving process to prepare aerogels, simple evaporation of solvent from the hydrogels under ambient conditions may cause significant shrinkage or even form solid films essentially without porosity.

#### 2.3.3. Freeze-Drying

In general, freeze-drying technique is a simple, more economic and environmentally friendly process to obtain aerogels with good porous structures. In this method, liquid in the wet gel is first frozen and thereafter dried by sublimation under low pressures. Thus obtained materials are also termed cryogels. These freeze-dried “cryogels” show a porosity of 80% at maximum and only half the inner surface of a comparable aerogel. Special precautions for freeze-drying are long aging periods to stabilize the gel body, a solvent exchange to provide a low expansion coefficient and high sublimation pressure, and the addition of salts to achieve low freezing rates and freezing temperatures [3]. Compared to aerogels prepared with supercritical CO_2_ drying, cryogels show more macroporous structures with larger shrinkage and Brunauer–Emmett–Teller (BET) value. In constrast, compared to aerogels synthesized by hot-drying or vacuum-drying, cryogels show smaller shrinkage, narrower pore size distribution and smaller BET value [88,89]. Practically, the aerogels prepared from aqueous solution or regenerated from water (e.g., cellulose, PI, PVA, pectin) are usually dried by this method.

**Figure 3:**
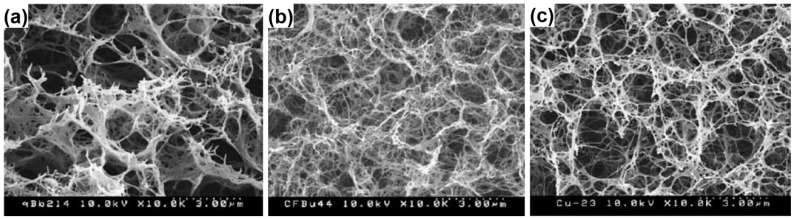
SEM images of 1% cellulose aerogel by (**a**) regular freeze drying; (**b**) solvent exchange drying; and (**c**) rapid freeze drying. Reproduced with permission of Ref. [90] (Copyright Elsevier 2004).

Cellulose aerogels prepared by different drying methods were investigated [90]. The regular freeze-dried aerogels (Figure 3a) had highly porous structure consisting of microfibrillar networks, but close examination reveals that the fibrils are severely coagulated to form film-like masses, especially at lower cellulose concentrations. In contrast, the solvent-exchange drying of water-methanol-*t*-butyl alcohol was effective in preserving the network structure (Figure 3b), resulting in aerogels consisting of finer fibrils of about 50 nm wide. The nitrogen adsorption surface area of the solvent-exchange dried aerogels ranged in 160–190 m^2^·g^−1^, is about twice of those of regular freeze-dried materials. Rapid freeze drying by metal plate-contact technique provided partial preservation of the original network structure, giving asymmetric porous structures as shown in Figure 3c. The surface area of regular freeze-dried material (70–120 m^2^·g^−1^) was about half of those of solvent-exchange materials, and also was more strongly dependent on starting cellulose concentration.

#### 2.3.4. Other Drying Methods

Other techniques such as microwave drying and vacuum drying have also been applied to obtain aerogels with high surface area and porosity [80,81,82]. Microwave drying is a time-saving method to prepare mesoporous and 3D interconnected macroporous aerogels. Aerogels prepared by this method show similar structures to those prepared by freeze-drying, but with more small-sized macropores. Vacuum drying is an energy-conserving process in which materials are dried under a reduced pressure, which needs less heat for rapid drying [91,92]. For example, microporous PI aerogels were prepared with BET surface area up to 1454 m^2^·g^−1^ and a narrow pore size distribution in the range from 5 to 6 angstrom via vacuum drying [91].

Among various drying methods, supercritical drying technique is the most effective method to prevent the drying shrinkage or collapse of mesopores and obtain well-defined structures. However, the long-time solvent exchange, the involvement of massive organic solvent, high temperature and pressure used in the drying process make it a time-consuming and high-cost method, which severely limit their practical applications. On the contrary, freeze-drying technique is a much easier, more economic and environmentally friendly method. Although the direct freeze drying is simple, it is heavily dependent on freezing rate and concentration of precursors in the wet gel. Ordered porous aerogels can only be obtained by elaborately controlling the freeze-drying process. Ambient pressure drying is designed to dry the wet gel at ambient pressure, which can be applied in large-scale industrial production, whereas it may result in environment pollution for the evaporation of solvents. For microwave drying and vacuum drying, they usually produce large pores or even induce the collapse of materials. Therefore, the drying methods have a great influence on the structure, surface area, porosity, and pore volume of aerogels and proper drying methods should be considered according to the practical requirements.

### 2.4. Carbonization of Organic Aerogels

Carbon aerogels are mostly obtained by pyrolysis, at temperatures above 600 °C, of organic aerogels. In this way, the organic aerogels transform to an electrically conductive carbon network.

Generally, carbonization of the dried aerogels is carried out by heating the sample in flowing N_2_ or Ar at a temperature between 600 and 2500 °C [93,94]. During carbonization, the aerogel loses oxygen and hydrogen functionalities, thus resulting in an enrichment in carbon and a highly pure carbon structure. Interestingly, the electrical conductivity of the samples increases significantly during this transformation, as indicated by the common appearance of a broadband, semi-metal-like infrared absorbance. Variations in the pyrolysis conditions cause significant changes in the properties of carbon aerogels. Higher pyrolysis temperatures tend to reduce the surface areas of the carbon aerogels, and their electrochemical double layer capacitance also decreases. The minor decrease in the surface area with increasing pyrolysis temperature is limited to temperature above 600 °C, whereas increasing pyrolysis temperature while under 600 °C increases the surface area [95]. However, the carbon aerogels may not be electrically conductive unless carbonized above 750 °C. The electrochemical double layer capacitance also exhibits a maximum between 800 and 900 °C [96]. At low carbonization temperatures, the macropore volume decreases and the mesopore volume increases due to the shrinkage of the material, whereas the micropore volume and surface area are enlarged due to the evolution of gases during carbonization. At higher carbonization temperatures, all these parameters tend to decrease. For example, carbon aerogels carbonized at 1200 °C are significantly denser than those carbonized at 600 °C [97]. The temperature required for complete graphitization of the carbon aerogel may exceed ≥2000 °C, but aerogels pyrolyzed at ~1050 °C contain separated graphitic structures, which has also been reported by others based on XRD patterns [98]. Therefore, for achieving better electrochemical performance, the aerogels are normally carbonized between 600 and 900 °C, whereas higher carbonization temperature (above 1000 °C) is employed to realize graphitization of the carbon aerogels for better electrical conductivity. In addition, activation of the aerogel can be carried out by its gasification with steam or CO_2_ at between 800 and 900 °C for varying time periods. As a result, the surface area, pore volume, and pore size distribution of carbon aerogels are tunable in a wide spectrum related to the synthesis and processing conditions.

## 3. Structures and Properties of Polymer/Carbon-Based Aerogels

### 3.1. Polymer Aerogels

Unlike silica aerogels being fragile and hygroscopic, polymer aerogels usually exhibit better mechanical and environmental stability for a variety of applications, especially in extreme environments such as aerospace. These organic aerogels can be produced with very little shrinkage during the processing and have thermal conductivity comparable to silica aerogels of similar density (only 14 mW·m^−1^·K^−1^) for aerogels with density of 0.1 g·cm^−3^) but are much stronger with compressive modulus ranging from 1 to 5 MPa [99]. Types of Polymer aerogels are diversified with different types, including cellulose-based, resin-based, PI-based and PVA-based aerogels, exhibiting diverse structures and properties. Depending on the polymer type and fabrication conditions, the structure of polymer aerogels can be changed from colloidal-like nanoparticles to nanofibrillar/microfibrillar networks and even to sheet-like skeletons. In addition, structural parameters such as pore size, ordering, and even pore shape (regular or irregular) will strongly affect the final macroscopic performance of the aerogel materials.

#### 3.1.1. Cellulose-Based Aerogels

Cellulose-based aerogels are one of the “oldest” aerogels and the first organic aerogels, which were invented accompanying the birth of silica aerogels [100]. Commonly, the cellulous gels are formed by dissolution and regeneration of cellulose in an aqueous or organic solvent [101,102]. Considering the nanoscale lateral dimensions of nanocellulose, it can potentially generate porous structures with ultralow density (less than 5 mg·cm^−3^) and exceptionally high porosity (over 99%). The aerogels have highly porous networks consisting of fibrils ranging from tens of nanometers to tens of micrometers [103,104]. The open 3D structure can transform from nanofibrillar to microfibrillar networks and even to sheet-like skeletons by changing the initial cellulose concentration and drying methods [90,105]. In another case, cellulose aerogels are composed of “globules” attached to each other and their size decreases with the increase of cellulose concentration [106,107]. Moreover, Cai *et al.* [108] demonstrated that ultralight pure natural aerogel microspheres can be fabricated using cellulose nanofibrials directly. The aerogel microspheres are highly porous with bulk density as low as 0.0018 g·cm^−3^ and pore size ranging from nanometers to micrometers. Covalent crosslinking between the native nanofibrils and cross-linkers made the aerogel microspheres very stable even in a harsh environment.

The use of naturally existing substances (e.g., biomass) is an emerging approach for the production of new functional nanoporous aerogel materials. The production of aerogels from inexpensive biomass-derived precursors has recently gained a lot of interests both academically and commercially because of the range of economic/process/chemistry advantages offered by such synthetic approaches. A variety of raw materials, such as paper waste [109], bamboo leaf [110], rice straw [111], and jute fibers [112] have been utilized to fabricate cellulose aerogels. Nguyen *et al.* provided a cost effective and scalable recipe for fabricating biodegradable cellulose aerogels from paper waste. The cellulose aerogels are macroporous with a low density of about 0.04 g·cm^−3^ and thermal conductivity from 0.029 to 0.032 W·m^−1^·K^−1^. Cellulose aerogels were also synthesized from bamboo leaf in acidic conditions, which exhibit large specific surface area (547.2 m^2^·g^−1^) and low thermal conductivity (0.024 W·m^−1^·K^−1^) [110]. An ultra-light (1.7 to 8.1 mg·cm^−3^) and ultra-porous (99.5% to 99.9%) aerogel has been assembled from cellulose nanofibrils that were defibrillated from rice straw cellulose at a yield of 96.8%. Furthermore, with both hydrophilic hydroxyls and hydrophobic pyranose rings, amphiphilic cellulose nanofibrils aerogels allow the uptake of both polar and nonpolar liquids, in contrast to the hydrophobic carbon-based aerogels and the hydrophilic inorganic oxide aerogels [111].

**Figure 4:**
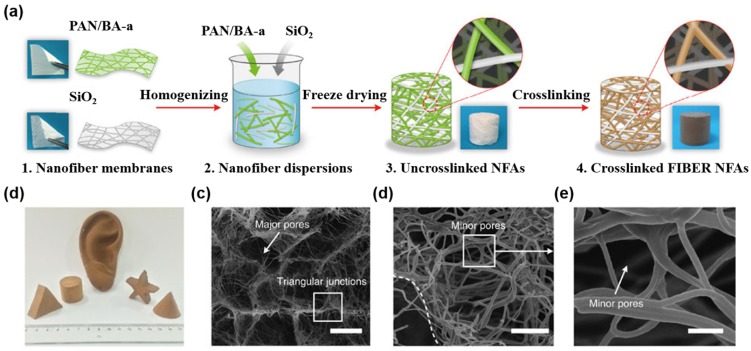
Design, processing and cellular architectures of FIBER nanofibrous aerogels. (**a**) Schematic showing the synthetic steps. (1) Flexible PAN/BA-a and SiO_2_ nanofiber membranes are produced by electrospinning. (2) Homogeneous nanofiber dispersions are fabricated via high-speed homogenization. (3) Uncrosslinked nanofibrous aerogels (NFAs) are prepared by freeze drying nanofiber dispersions. (4) The resultant FIBER NFAs are prepared by the crosslinking treatment. (**b**) An optical photograph of FIBER NFAs with diverse shapes. (**c**–**e**) Microscopic architecture of FIBER NFAs at various magnifications, showing the hierarchical cellular fibrous structure. Reproduced with permission of Ref. [113] (Copyright Nature Publishing Group 2014).

As mentioned above, nanofibrous aerogels with high continuity and an open-cell cellular structure were fabricated from cellulosic materials, including bacterial cellulose fibrils, cellulose nanocrystals and lignocellulose, *etc*. However, the inherent limits on the diversity of bulk materials, combined with the lack of precise control of the physicochemical and mechanical properties, present major challenges in the synthesis of nanofibrous aerogels that must be addressed before their extensive practical applications. In contrast, electrospun nanofibers, which combine the merits of robust mechanical strength, low density, fine flexibility, extremely high aspect ratio and ease of scalable synthesis from various materials (polymer, ceramic, metal, carbon and so on), hold great promise as an exceptional nanoscale building block for constructing macroscopic nanofibrous aerogels. Recently, Si and coworkers presented a robust methodology for creating superelastic nanofibrous aerogels with a hierarchical cellular structure that consists of bonded nanofibers, which are called “fibrous, isotropically bonded elastic reconstructed” (FIBER) nanofibrous aerogels (Figure 4) [113]. The intrinsically lamellar deposited electrospun nanofibers are reconstructed into 3D elastic bulk aerogels with tunable densities and desirable shapes on a large scale. The resulting FIBER nanofibrous aerogels exhibit the integrated properties of extremely low density (minimum of 0.12 mg·cm^−3^), super recyclable compressibility and multifunctionality in terms of combining thermal insulation, sound absorption, emulsion separation and elasticity-responsive electric conduction, all originating from the synergistic effect of hierarchical cellular fibrous networks and well-bonded nanofibers. In dramatic contrast to the brittle nature of traditional colloidal aerogels, the FIBER nanofibrous aerogels show superelastic mechanical properties, which can bear a compressive strain (ε) as high as 80% and can recover their original volume after the release of the stress (σ). The maximum σ was 10.6 kPa for the first elastic regime and 36.1 kPa at 80% strain. These values were significantly higher than those of other fibrous aerogels with similar densities. In addition, no significant decrease in the stiffness or strength was observed for FIBER nanofibrous aerogels by 1000 cyclic compressions. The successful synthesis of such fascinating materials may provide new insights into the design and development of multifunctional nanofibrous aerogels for various applications.

#### 3.1.2. Polysaccharide-Based Aerogels

Polysaccharides are regarded as key ingredients for the production of bio-based materials in life sciences (e.g., food, cosmetics, medicaldevices, pharmaceutics). The biodegradability and biocompatibility of these biopolymers, coupled to the large variety of chemical functionalities they encompass, make them promising carriers for drug delivery systems. In nature, polysaccharides are known to self-associate or self-order into particular structures, physical forms, or shapes (e.g., the starch granule, plant structures). Significantly for materials chemistry, polysaccharides can also self-associate upon inducement of an aqueous gel state. This provides the materials chemist the opportunity to exploit this ability for the preparation of new porous aerogel and xerogel materials. Polysaccharide-based aerogels are highly porous (ε = 90%–99%) and lightweight (ρ = 0.07–0.46 g·cm^−3^) with high surface area (S_BET_ = 70–680 m^2^·g^−1^), able to provide enhanced drug bioavailability and drug loading capacity [114,115]. Alternatively, the resulting aerogel materials can also meet the performance criteria for other emerging niche markets, such as catalysts, catalyst supports or adsorbents, providing a new opportunity to obtain useful materials from one of the less energy-intensive sources of biomass.

In the late 1990s, Glenn *et al.* and Te Wierik *et al.* independently demonstrated that the low surface area of native starch could be expanded to generate xerogel materials with specific surface areas (S_BET_) of between 25 and 145 m^2^·g^−1^ depending on preparative route [116,117]. Clark and coworkers demonstrated the potential of cornstarch (≈73% amylopectin) in the production of low density, high surface area starch xerogels (S_BET_ ≈ 120 m^2^·g^−1^) for applications in normal-phase chromatography separations [118]. Recently, this work was extended to the microwave-assisted preparation of high surface area (S_BET_ > 180 m^2^·g^−1^), highly mesoporous starch-derived materials (Vmeso > 0.6 cm^3^·g^−1^; >95% mesoporosity) [119]. This research demonstrated that the key to the formation of the porous polysaccharide form in starch was the generation of polysaccharide gel.

Chitosan is a biopolymer obtained from the deacetylation of chitin, which is itself extracted from shells of crustacean and mollusks such as shrimps, crabs and squids. Chitin is the second-most-abundant polysaccharide next to cellulose and it is produced in approximately one hundred billion tons per year. The interest for chitosan stems from its applications in biomaterials, drug-delivery systems, food additives, water clarification, and as support for cells and enzymes [120]. Chitosan can form either chemical or physical hydrogel. Chemical hydrogels are formed by irreversible covalent links, as in covalently crosslinked chitosan hydrogels. Physical hydrogels are formed by various reversible links, as in ionically crosslinked hydrogels and polyelectrolyte complexes, or in entangled gels. The latter are formed by solubilization of chitosan in an acidic aqueous medium, and precipitation in an alkaline solution, which is the simplest way to prepare a chitosan hydrogel. The choice of the cross-linker and its concentration should be a compromise between aerogel stability and the required open porosity and homogeneity [121]. In general, chemical gels allow a processing with better control of the porous structure and swelling behavior than with physical gels, but at expense of higher raw materials and processing costs and more complex chemical characterization. After solvent exchange to an alcohol, chitin hydrogels can then be supercritically dried to obtain chitin aerogels of high porosity, high surface area and low density with changes in value depending on the chitin concentration and the alcohol solvent used in the original wet gel. For a given degree of deacetylation, the type of chitin affects the distribution of the residual acetylated glucosamines, the crystallinity of the polymer and its gelling properties. Generally, aerogels with higher surface area are obtained from hydrogels of chitosan obtained by deacetylation of α-chitin from crab shell than by deacetylation of β-chitin from squid pen [122].

The utilization of polysaccharides in the preparation of aerogel materials represents an interesting alternative to the materials prepared through conventional polymerization and cocondensation techniques. Furthermore, the use of polysaccharides derived from biomass potentially adds value to inexpensive and typically waste products from industry such as the food sector.

#### 3.1.3. Resin-Based Aerogels

RF-based aerogels were first described by Pekala through the polycondensation of resorcinol (R) with formaldehyde (F) in an aqueous environment using Na_2_CO_3_ as a catalyst [15]. The polymerization reaction consists of two steps: (1) addition reaction between R and F to form hydroxymethyl resorcinol monomers; (2) –CH_2_– or –CH_2_OCH_2_– bridging polymerization between monomers, producing water/formaldehyde or water. Continuous polymerization will produce RF clusters which further crosslink throughout the whole colloid system to gelate. The as-prepared RF aerogels consist of interconnected colloidal-like particles with diameters of approximately 10 nm and form the surface functionalized polymer “clusters” with a low density below 0.1 g·cm^−3^. The Na_2_CO_3_-catalyzed RF aerogels and corresponding sol-gel process are most widely adopted till now, because of the good formability, easy microstructure-controllability and adjustable properties [25,82,123,124].

However, the rate of these reactions is very slow at room temperature. Normally, a multiple stage heating process is used to accelerate the gelation process. Besides, catalysts were also applied to accelerate the polycondensation process [123,125,126,127,128,129,130,131]. The acid-catalyzed route, e.g., acetic acid, hydrochloric acid (HCl), oxalic acid or para-toluenesulfonic acid, was reported for the preparation of RF aerogels. Compared with the typical base-catalyzed RF aerogels, acid-catalyzed processes are more time-efficient. The usage of HCl as catalyst for the preparation of RF aerogels leads to organic aerogels in very short gelation times, varying from a few seconds to minutes [128]. The obtained RF aerogels have a 3D network that consists of approximately monodispersed spherical-like particles in the range of 700–1500 nm. On the other hand, the RF aerogels prepared by base-catalyzed route show a narrow pore size distribution (PSD) with the maximum position located between 10 and 20 nm, while the pores of RF synthesized by acid-catalyzed route are 10 times larger with a broader PSD [132]. Wu *et al.* [133] prepared phenol-furfural aerogels by dividing the sol-gel polymerization into two steps, *i.e.*, the formation of gel precursors by pre-polymerizing phenol and furfural with NaOH as the catalyst and subsequently the gelation of the precursors with HCl as the catalyst. It was found that the crosslinking density of the gel precursors, controlled by the catalytic time of NaOH, exerts a great influence on the yield, drying shrinkage, bulk density and texture of the aerogels obtained. An appropriate increase in the crosslinking density of the gel precursors helps to increase the yield and mesoporosity, leading to an increase of the bulk density. However, the organic aerogels obtained with NaOH-catalyzed pre-polymerization process experience a larger drying shrinkage than those without NaOH-catalysts. In addition, the application of ammonium carbonate as catalyst for the preparation of RF-aerogels leads to organic aerogels without metallic impurities in contrast to the conventional catalysts like sodium carbonate. The particle size and density of the synthesized RF aerogels are 0.15–4 μm and 0.37–0.42 g·cm^−3^, respectively [76].

A new class of phenolic resin, namely polybenzoxazine, is recently developed. Polybenzoxazine has excellent molecular design flexibility that allows the properties of the cured materials to be controlled for various applications. Polybenzoxazine has many advantageous characteristics compared with traditional phenolic resin, such as high mechanical strength, near-zero shrinkage upon polymerization of its monomer, and high char yield [134,135]. Interestingly, ring-opening polymerization of benzoxazine in solvents leads to gelation and formation of 3D polymer networks [136,137]. Lorjai *et al.* first reported the synthesis of polybenzoxazine gels by thermally-induced ring opening polymerization of benzoxazine in xylene at 130 °C [138]. The subsequent carbon aerogels prepared by the carbonization of polybenzoxazine aerogels exhibited a microporous structure with pore diameters less than 2 nm, the densitiy of 300 kg·m^−3^, and surface area of 384 m^2^·g^−1^, respectively. The most prominent characteristic of the polybenzoxazine-derived carbon aerogels is high proportion of micropore volume to micropore surface area, as compared with organic aerogels obtained from other precursors. Recently, Mahadik-Khanolkar *et al.* published a new method for preparation of polybenzoxazine gels at room temperature by using hydrochloric acid as the catalyst of cationic ring opening polymerization [139]. The scanning electron microscopy images revealed that the corresponding aerogels consisted of spherical polymer particles as the building block and that the diameter of spherical particles reduced with an increase in the concentration of benzoxazine in solution. Time-efficient gelation of benzoxazine using p-toluenesulfonic acid as the catalyst in several solvents and controllable pore structure formation in the resultant polybenzoxazine aerogels are reported by Gu and coworkers [140]. All of the aerogels synthesized in different solvents were found to be mesoporous materials, and the skeletal frameworks of polybenzoxazine aerogels can be tuned using appropriate solvents and suitable polymerization temperature.

#### 3.1.4. Polyimide-Based Aerogels

**Figure 5:**
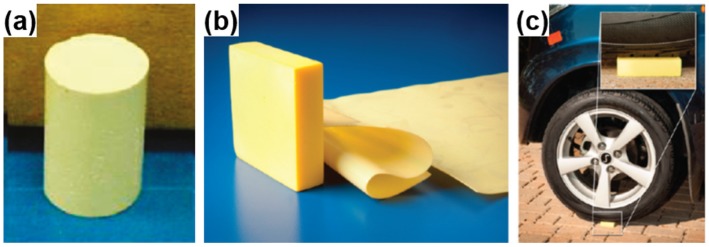
(**a**) Octa(aminophenyl)-silsesquioxane (OAPS) cross-linked polyimide aerogels; (**b**) polyimide aerogels cross-linked with 1,3,5-triaminophenoxybenzene (TAB) fabricated as flexible thin films or molded to a net shape; and (**c**) demonstrating strength of the aerogel by supporting the weight of a car. Reproduced with permission of Ref. [99] (Copyright American Chemical Society 2012).

Polyimides are widely used in industries for their high performance, such as ultralow dielectric constant, high mechanical strength and thermal stability at high temperatures [16,85,99,141,142,143,144,145]. Therefore, it would be ideal to design PI aerogels for use as highly porous, lightweight and high-temperature stable materials. PI aerogels prepared from aromatic dianhydrides and diamines, have been synthesized by gelation of chemically or thermally imidized PIs in dilute solutions, followed by drying. The PI aerogels exhibit a rich variety of nanoporous structures, such as crisp fragments [83], loose or close clusters of polymer fibers [99,143,144,146] in the range of tens of nanometers to several micrometers. It was found that the morphologies are highly dependent on the solvent interactions during polymerization, as well as the chain rigidity and chain length of monomers and crosslinkers. One merit of PI aerogels is their excellent mechanical properties, which meets the high standard for applications in aerospace. PI aerogels can be produced with very little shrinkage during the processing and have high compressive modulus ranging from 1 to 5 MPa, much higher modulus than typical polymer reinforced silica aerogels with similar density exhibiting compressive modulus ranging from 0.1 to 2 MPa. Additionally, PI aerogels are light yellow to orange yellow in appearance, which can be tailored into different shapes, as shown in Figure 5. Thin films (nominally 0.5 mm) of the aerogels from certain formulations are flexible to be rolled or folded backward and completely recover with no evidence of cracking or flaking. Thicker parts such as the 6.5 cm × 6.5 cm × 1.3 cm rectangular prism shown in Figure 5b are rigid and strong, even able to support the weight of a car, as seen in Figure 5c [99].

Imidization is essential for the formation of PI aerogels during gelation, which can be induced either chemically or thermally. The aerogels prepared by thermal imidization exhibited very little shrinkage with a porosity of up to 90%. However, the gels tended to redissolve during thermal imidization and reform upon cooling, obviating the need to keep the gels in molds. This is not conducive to fabrication of freestanding films using roll-to-roll processing, and also suggests that part of the amic acid hydrolyzes during thermal imidization, which breaks up the network structure [83,99]. On the other hand, PI aerogels prepared by chemical imidization exhibit various structures and properties, which is strongly related to the choice of crosslinking agents. A series of octa(aminophenyl)-silsesquioxane (OAPS) crosslinked PI aerogels were prepared by Guo and coworkers [143]. The morphology of as-prepared PI aerogels resembles bundles of polymer fibers tangled together with fiber diameters in the range of 15–50 nm. The aerogels can be produced with very little shrinkage during the processing and have thermal conductivity comparable to silica aerogels with similar density (only 14 mW·m^−1^·K^−1^ for aerogels with density of 0.1 g·cm^−3^) but are much stronger with compressive modulus ranging from 1 to 5 MPa. Most notably, these aerogels can be fabricated as thin films that are flexible and foldable, being stable up to 400 °C for short-term exposure as well. PI aerogels using 1,3,5-triaminophenoxybenzene (TAB) as the crosslinker were also reported [16]. The as-prepared PI aerogels show a low dielectric constant value at 1.08 and high compressive moduli of 4–8 MPa (40 times higher than silica aerogels at the same density). In comparison to PI aerogels crosslinked using OAPS, the one crosslinked with TAB has a density about 26% higher, but modulus increases by a factor of 4 and surface areas are also significantly higher for these TAB crosslinked aerogels.

**Figure 6:**
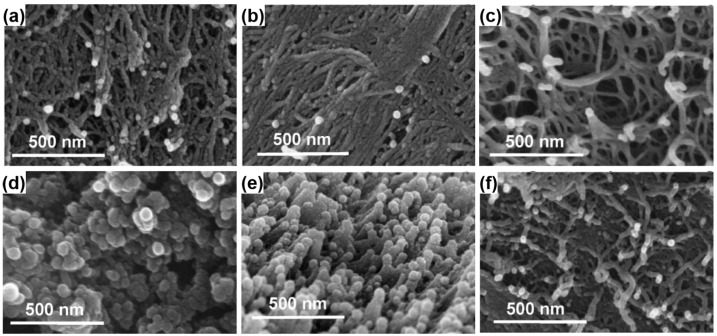
SEM images of PI aerogels synthesized from (**a**) ODA/BPDA; (**b**) PPDA/BPDA; (**c**) DMBZ/BPDA; (**d**) ODA/BTDA; (**e**) DMBZ/BTDA; and (**f**) PPDA/BTDA. Reproduced with permission of Ref. [99] (Copyright American Chemical Society 2012).

The property of PI aerogels could be affected by the backbone structures, thus aerogels with different combinations of diamine and dianhydride are examined [99]. A series of TAB crosslinked aerogels derived from three different diamines (*i.e.*, *p*-phenylene diamine (PPDA), 2,2′-dimethylbenzidine (DMBZ), and 4,4′-oxydianiline (ODA)) and two different dianhydrides (*i.e.*, benzophenone-3,3′,4,4′-tetracarboxylic dianhydride (BTDA) and biphenyl-3,3′,4,4′-tetracarboxylic dianydride (BPDA)) were prepared and studied. BPDA, PPDA, and DMBZ produces a rigid backbone in the main chain of PI, leading to higher glass transition temperatures, whereas ODA and BTDA provide flexible linking groups between phenyl rings, thus resulting in less rigid structures. Interestingly, the pore structures observed by SEM images shown in Figure 6 for the aerogels produced using different diamines and dianhydrides are quite dissimilar. Aerogels derived from BPDA (Figure 6a–c) all appear as collections of polymer strands ranging from 30–50 nm in thickness. The combination of BTDA and ODA (Figure 6d) produces aerogels that are the most similar to silica aerogels with clusters of 50–100 nm particles loosely connected together. The aerogels composed of BTDA and DMBZ (Figure 6e) also appears as clusters of nanoparticles with more uniform and smaller size (∼50 nm). Physical and mechanical properties of TAB crosslinked aerogels polymerized from different dianhydrides and diamines also vary a lot. It was found that aerogel properties, such as density and mechanical properties, were highly dependent on the shrinking degree during gelation. Aerogels made using a combination of BPDA and DMBZ shrank the least and therefore had the lowest density, whereas using BTDA and ODA led to greater shrinkage and higher densities. Aerogels made using ODA also tended to be less brittle, whereas DMBZ-based aerogels tended to decompose at lower temperatures. The PI aerogels have a high onset decomposition temperature over 600 °C using PPDA as the backbone diamine with excellent tensile strengths ranging from 4 to 9 MPa which can support the weight of a car. Furthermore, it was found that using mixtures of diamines led to combinations of improved properties. For example, 100% ODA gives flexible aerogels that are hydrophilic while 100% DMBZ gives more hydrophobic aerogels that are brittle. A mixture of 50% ODA and 50% DMBZ used in conjunction with BPDA gives polyimide aerogels with better moisture resistance and flexibility.

Different from the methods of preparing PI aerogels via polycondensation of dianhydrides and diamines, Leventis *et al.* [146] prepared PI aerogels by ring-opening metathesis polymerization of a norbornene end-capped diimide. The as-prepared PI aerogels retain high porosity (50%–90%, *v*/*v*), high surface area (210–632 m^2^·g^−1^, of which up to 25% is traced to micropores), and uniform pore size distribution in the mesoporous range (20–33 nm). The skeletal framework consists of primary particles of 16–17 nm in diameter, assembling to form secondary aggregates of 60–85 nm in diameter. The aerogels show a thermal conductivity of 0.031 W·m^−1^·K^−1^ and high mechanical compressive strength of 168 MPa. Hence, they are reasonable multifunctional candidates for thermal/acoustic insulation materials.

Overall, PI aerogels, one kind of the emerging aerogels, have attracted great interests for their unique thermal, mechanical and dielectric properties, which are ideal candidates for thermal or electrical insulation materials, especially in aerospace areas.

#### 3.1.5. PVA-Based Aerogels

Poly(vinyl alcohol) is a highly hydrophilic and water-soluble polymer, synthesized by partial or complete hydrolysis of poly (vinyl acetate). The low toxicity, high water content, good mechanical properties and biocompatibility of PVA hydrogels make them widely used in various fields, such as drug delivery, contact lenses, wound bandages, tissue engineering and cell immobilization [17]. The preparation of PVA aerogel is usually simple and not diversified. Typically, an appropriate amount of PVA is dissolved in deionized water at 70–80 °C under vigorous stirring until the polymer is completely dissolved. Then, PVA hydrogels can be fabricated via different techniques, such as the freeze-thaw inducement of crystallization, acid-catalyzed dehydration, irradiation, radical production and chemical crosslinking. Subsequently, the hydrogels are usually freeze-dried to form aerogels. One advantage but also the limitation of PVA aerogels is that it can be fabricated in aqueous media followed by facile freeze-drying method, which is a green and time efficient approach to fabricate aerogel materials.

#### 3.1.6. Polymer-Based Hybrid Aerogels

In order to strengthen the nanostructure and suppress the collapse of nanopores of polymer aerogels, nanofillers, such as SiO_2_, clay, cellulose nanofibrils (CNFs), CNTs, and graphene or graphene oxide (GO) are usually incorporated into the polymer matrices to prepare nanofiller-polymer hybrid aerogels. The hybrid aerogels always exhibit enhanced mechanical or thermal properties as well as multifunctions as compared with pure polymer aerogels.

##### Clay-Polymer Hybrid Aerogels

In general, clay is introduced into aerogels to enhance the rigidity and flame retardancy properties of the aerogels. Sodium-montmorillonite (MMT) was incorporated into cellulose aerogels to obtain composite aerogels, which show a honeycomb-like porous structure and superior functionalities. The modulus of cellulose increases from 25.8 kPa and 2.13 MPa to 386 kPa and 3.86 MPa for the isotropic and anisotropic aerogels, respectively [34]. Additionally, the inclusion of MMT increased the heat endurance of the aerogels dramatically up to 800 °C while the mechanical properties were retained up to 300 °C with shape retention functions. A series of PVA/clay aerogel composites were investigated by Chen and coworkers, which were crosslinked either by radiation or chemical bonding to show a layered microstructure [17,36,37]. Furthermore, they indicate that crosslinking can increase the compressive modulus of aerogels and the strengthened PVA/clay aerogels possess very low flammability. PI/clay aerogel composites were also produced by freeze-drying of a PAA ammonium salt/clay precursor suspension, exhibiting layered structures and low densities in the range of 0.04–0.09 g·cm^−3^ [40].

##### CNT/CNF-Polymer Hybrid Aerogels

Introduction of CNTs into polymer aerogels can improve the electrical or thermal conductivity of the aerogels. Multi-walled carbon nanotubes (MWCNT)/RF composite aerogels were prepared by sol-gel polymerization of RF monomers in an aqueous suspension of CNTs, followed by supercritical CO_2_ drying [23]. MWCNT can form a homogeneous uniform dispersion in aerogels at low concentrations, leading to a homogeneous material at the nanometer scale. The relative thermal conductivity increased slightly with the increasing MWCNT loading. The thermal conductivity of the composite gel exhibited 27% higher than that of the corresponding pure RF gel by incorporation of 1.04 vol % MWCNT. Due to the high surface area and aspect ratio, cellulose nanofibrils can easily form an entangled web-like structure to be used in a wide range of applications, such as antibacterial agents, thermal insulation and oil absorbents. Bringing CNFs into PVA aerogels could also lower the density (<15 kg·m^−3^) and increase the porosity (>98%) of the aerogels. More interestingly, the PVA/CNF aerogel became superhydrophobic and superoleophilic after being treated with methyltrichlorosilane [33]. The silane-treated, cross-linked PVA-CNF aerogels exhibit excellent absorption performance for various types of oils, organic solvents and heavy metal ions (e.g., Pb^2+^, Hg^2+^). Furthermore, these silane-treated PVA-CNF aerogels demonstrate excellent elasticity and mechanical durability. A ternary hybrid aerogel containing PVA, CNF and MWCNT was also prepared by the same group, which showed low density (<0.031 g·cm^−3^), high surface area (160–200 m^2^ g^−1^), and very low thermal conductivity (<0.031 W m^−1^ K^−1^) [39].

##### GO-Polymer Hybrid Aerogels

GO has drawn a great deal of attention as a reinforcing nanofiller in polymer composites due to its low cost, unique two-dimensional honeycomb layered structure, and excellent mechanical properties (fracture stress ~63 GPa) [147,148]. Compared with CNTs, nanoplatelets have a geometry that can offer isotropic reinforcement in more than one direction, and exhibit more effective reinforcement in polymer composites. Moreover, with numerous oxygen functional groups on the surfaces, GO can be readily dispersed in water to form a stable colloidal suspension and also form strong interfacial bondings with the polymer matrix [149]. As a consequence, GO can act as the “super gelator” in aqueous solution with a critical gelation concentration <0.5 wt %, thus obviously accelerating the gelation process to reduce both the drying shrinkage and aerogel density [150]. Guo *et al.* [24] prepared RF composite aerogels by a simple sol-gel polymerization method using graphene oxide as an anti-shrinkage additive. Morphological studies showed that GO can be well dispersed in the RF matrix owing to the good compatibility between the two elements, which effectively strengthen the RF skeleton. The linear shrinkage decreases progressively from 28.3% to 2.0% with GO loadings increasing from 0 wt % to 2 wt %, while the density decreases from 506 to 195 kg·m^−^^3^ simultaneously. This work offers a new method for the inhibition of aerogel shrinkage and broadens the application scope of GO as well. The gelation behavior of aqueous dispersions composed of GO and PVA was investigated through rheological study [151]. Strikingly, it was found that small amplitude oscillatory shear can induce the gelation of graphene dispersions and the rheological properties measured after the completion of SAOS-induced gelation was highly related with the density and the electrical conductivity of graphene aerogels. The as-prepared ultra-light aerogel exhibited low density of 4.0 mg·cm^−3^, low surface resistivity of 6.6 U·sq^−1^, high specific area of 1069 m^2^·g^−1^, and high recoverable strain of 94% in compression. Moreover, the glass transition temperature of PVA in the aerogel was 49 K higher than that of pure PVA. Superhydrophobic and superoleophilic graphene/polyvinylidene fluoride (G/PVDF) aerogels were prepared by the solvothermal reduction of GO/PVDF mixed dispersions [32]. The as-prepared aerogel shows high specific surface area, eminent adsorption capacity for oils and organic solvents, superior water repelling ability, and excellent adsorption recyclability. Therefore, this kind of aerogel is a promising material for oil–water separation, oil spill cleanup and recovery of organic solvents.

In addition to those nanofillers mentioned above, various other inorganic components, such as metal or metal oxides, have been incorporated into polymer aerogels to meet the demand for multi-functions. For example, silica was incorporated into PVA aerogels to enhance the thermal stability and lower the flammability of the aerogels [36]. Flexible and porous zinc oxide bionanocomposite foams were prepared by controlling the hydrolysis and solvothermal crystallization in which cellulose was used as a template, showing excellent antibacterial activity [152].

### 3.2. Carbon Aerogels

Carbon aerogels are composed of nanostructured porous carbon materials in which the pores are filled with gas. Carbon aerogels have been extensively studied as they combine beneficial adsorption properties and structural stability with high thermal stability and, in principle, electronic conductivity, for potential applications in catalyst carriers, oils or organic solvents adsorption and energy storage [153,154]. One important feature of carbon aerogels is their ultralight nature, with the lowest density ever reported, 0.16 mg·cm^−3^ [155]. Besides being highly porous and lightweight, another advantage is the possibility to introduce electrical conductivity after being converted into carbon aerogels, thus allowing applications in electrical/electrochemical areas (e.g., batteries, supercapacitors, or conductive catalyst supports). Traditionally, carbon aerogels are fabricated from organic gels through controlled thermal annealing/carbonization under a non-oxidizing atmosphere (e.g., flowing N_2_ or Ar). More recently, it has been reported that biomass-derived carbon aerogels can be synthesized through the aqueous gelation and self-assembly of suitable biomass-derived precursors (e.g., polysaccharides, proteins, sugars). Additionally, with the development of novel carbon materials, graphene or CNT based carbon aerogels with ultralow density and excellent conductivity have been produced as emerging novel aerogel materials, which further expand the application fields of aerogel materials.

#### 3.2.1. Carbon Aerogels from Carbonization

The first carbon aerogel was born in 1989 by carbonization of RF aerogel. It is usually considered as a kind of highly-porous amorphous-graphite-based foam. The basic idea of preparing carbonized RF aerogel is to pyrolyze the high carbon-content precursor (RF aerogel) under high temperature (normally 800–1200 °C), ambient pressure and inert atmosphere. Like their parent organic aerogel systems, the carbonized gels have a network structure composed of interconnected nanosized primary particles. For conventional RF gel-derived carbon aerogels, micropores typically develop in the primary particles, whereas mesopores and macropores are generated as a result of the distances between the primary particles (originally occupied by solvent used in the synthesis of the parent organic gel). The porous dimensions of the parent organic gel do not automatically transfer directly into the carbon aerogel due to a variety of transformations (e.g., shrinkage, pore closure, aromatization/graphitization) may occur during the thermal annealing step. However, in principle, the amount of micropores and mesopores in these carbon aerogels can be dictated independently, a major advantage of carbon gels, through selection of the parent gel composition, drying method, curing time/temperature and, of course, the carbonization temperature.

**Figure 7:**
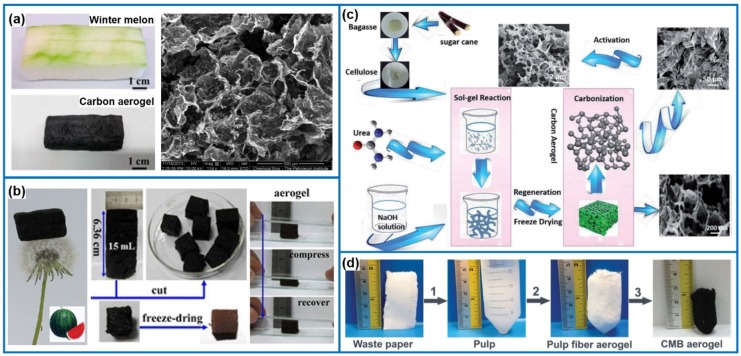
Carbon aerogels derived from different biomass: (**a**) Winter melon, reproduced with permission of Ref. [156] (Copyright American Chemical Society 2014). (**b**) Watermelon, reproduced with permission of Ref. [158] (Copyright American Chemical Society 2013). (**c**) Bagasse, reproduced with permission of Ref. [159] (Copyright The Royal Society of Chemistry 2014). (**d**) Waste paper, reproduced with permission of Ref. [160] (Copyright Wiley-VCH Verlag GmbH & Co. 2014).

More recently, biomass-based carbon aerogels were fabricated with the green chemistry concept, as shown in Figure 7. For example, Li *et al.* [156] fabricated carbon aerogels from the natural biomass, winter melon, via a hydrothermal and post-pyrolysis process (Figure 7a). The obtained carbon aerogel shows a low density of 0.048 g·cm^−3^, excellent hydrophobicity with a water contact angle of 135°, and selective adsorption for organic solvents and oils of 16–50 times its own weight. Similarly, watermelon was used as a renewable starting material to directly generate monolithic sponge-like carbon aerogel through a simple one-pot hydrothermal approach (Figure 7b) [157,158]. This approach does not need any additives as watermelons naturally contain water and carbohydrates. Directly cutting the native watermelon pieces could offer an easy shaping for hydrogels from 10 mL up to 1 L. After drying, low-density (*ca.* 0.58 g·cm^−3^), flexible, sponge-like aerogels made of nanospheres/nanofibers together with larger aggregated microparticles were obtained. In another case, highly porous carbon aerogels by using bagasse as a raw material were demonstrated in Figure 7c [159]. Macro and mesoporous carbon was first prepared by carbonizing the freeze-dried bagasse aerogel. Subsequently, microporous structure was created on the walls of the mesoporous carbon by chemical activation. Therefore, the as-prepared carbon aerogels with hierarchical porous structures exhibit high-energy storage ability as electrode materials in supercapacitors. Despite the fact that the direct approach based on fruit/vegetable is very simple and inexpensive, the control over the final morphology/porosity is quite limited and closely dependent on the native biomass. In addition, there might be some issues related to the competition with the food chain. Therefore, lightweight, hydrophobic and porous carbon microbelt aerogels and carbon fiber aerogels are produced via a facile route by using waste paper or cotton as the raw materials (Figure 7d) [160]. Importantly, the aerogel can adsorb a wide range of organic solvents and oils with a maximum sorption capacity up to 192 times of its own weight. Moreover, the aerogel also exhibits excellent recyclability, maintaining a high sorption capacity even after five cycles through distillation, burning or squeezing. The above synthesis strategies open a good pathway to employ abundant and renewable natural resources for manufacturing porous carbon materials, which may have great potentials for industrial applications and environmental protections.

The use of cellulose as a precursor for the preparation of aerogel and cryogels is well documented and has been discussed above. However, the transformation of cellulose aerogels into their carbonaceous or carbon equivalents has, until recently, not been significantly reported. Yu and coworkers have recently demonstrated the preparation of carbon aerogels using bacterial cellulose as the precursor polysaccharide for the preparation of bacterial cellulose aerogels and the thermal conversion to carbon aerogels [161,162]. Bacterial cellulose pellicles were first cut into the desired dimensions (e.g., 320 × 240 × 12 mm^3^) and purified, followed by freeze-drying to produce the bacterial cellulose aerogel. Thermal treatment under an inert (Ar) atmosphere between 700 and 1300 °C was employed to convert the polysaccharide aerogel into the carbon aerogel, denoted as the carbon nanofiber aerogel. After the pyrolysis step, the spatial volume of the obtained carbon nanofiber aerogel was only 15% of the original polysaccharide aerogel, and the density was found to decrease from 9–10 to 4–6 mg·cm^−3^ in the carbonized product. The resulting bacterial-cellulose-based carbon aerogels were composed of amorphous, turbostratic carbon nanofibers of 10–20 nm diameter, with an acceptable electrical conductivity of 20.6 S·m^−1^. Interestingly, the carbon nanofiber aerogels prepared using this approach displayed high flexibility, which is not commonly displayed by conventional low-density, high-porosity materials (e.g., silica-based aerogels). It was possible to manually compress these bacterial cellulose-derived carbon nanofiber aerogels with more than 90% volume reduction, followed by near-total recovery of the original volume after release of the force, demonstrating the unusually compliant and elastic features. Although the presented carbon nanofiber aerogels derived from bacterial cellulose possess interesting and potentially unique mechanical properties, it is not clear exactly why morphology of the bacterial cellulose does not lose with such a low carbonization yield upon carbonization, given the general thermal decomposition chemistry of polysaccharides.

To tune the electronic and surface chemical properties of carbon materials and improve their electrochemical performance, heteroatom-doped carbon aerogels were fabricated from bacterial cellulose. Specifically, P-doped, N,P-co-doped, and B,P-co-doped carbon nanofibers were successfully prepared by impregnating H_3_PO_4_, NH_4_H_2_PO_4_, and H_3_BO_3_/H_3_PO_4_ into the bacterial cellulose pellicle, respectively, followed by carbonization in an inert atmosphere at 800 °C [163]. Nitrogen-doped carbon nanofiber aerogels were also reported by annealing them in NH_3_ [164] or employing polyaniline coated bacterial cellulose as the precursor [165]. In the field of energy storage, Zhi *et al.* recently reported on the production of carbon aerogels from bacterial cellulose as an anode in Li-ion batteries in conjunction with deposited SnO_2_ and Ge nanoparticles. Similarly, bacterial-cellulose-derived carbon nanofiber@MnO_2_ and nitrogen-doped carbon nanofiber aerogels were also employed as electrode materials in the development of asymmetric or all-solid state supercapacitors [166].

#### 3.2.2. Graphene/CNT Based Carbon Aerogels

Novel carbon aerogels such as CNT and graphene aerogels are theoretically considered as the best candidates to make ultralight yet elastic and conductive aerogels, given their marvelous mechanical strength, low density, fine elasticity, good electrical conductivity, and extremely high aspect ratio [167,168,169]. CNT aerogel sheets are the sole component of new artificial muscles that provide giant elongations of 220% and elongation rates of (3.7 × 10^4^)% per second, respectively, at operating temperatures from 80 to 1900 K [170]. Regardless of their ultralight density, the CNT or graphene aerogels are mechanically robust, which can support a counterpoise up to 1000 times of its own weight. More interestingly, some CNT or graphene aerogels exhibit superelasticity, which can be repeatedly compressed and recovers most of its volume after the release of compression. Besides, CNT aerogels have an electrical conductivity of 3.2 × 10^−2^ S·cm^−1^ that can be further increased to 0.67 S·cm^−1^ by a high-current pulse method without degrading their structures [171]. Therefore, this kind of novel carbon aerogels further expands the application fields of aerogel materials, including organic light-emitting displays, solar cells, sensors, novel electrodes and cold electron field emission.

**Figure 8:**
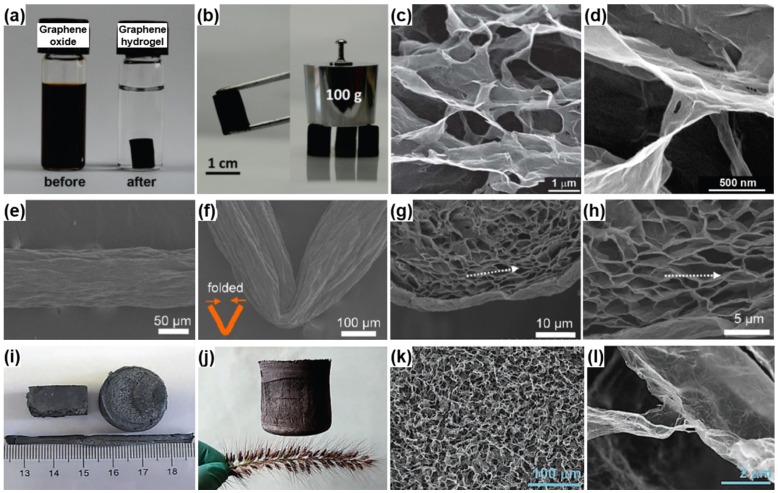
(**a**) Photograph of an aqueous GO dispersion (2 mg·mL^−1^) before and after hydrothermal reduction at 180 °C for 12 h; (**b**) photographs of a strong rGO hydrogel allowing easy handling and supporting weight; and (**c**,**d**) SEM images of the rGO hydrogel showing interior microstructures. Reproduced with permission of Ref. [50]; (Copyright 2010 American Chemical Society.) (**e**) SEM images of the surface of graphene aerogel fibers and (**f**) folded/stretched graphene aerogel fibers; (**g**,**h**) SEM images of fracture morphology of graphene aerogel fibers. The arrows indicate the alignment direction of graphene sheets. Macroscopic and microscopic structures of UFAs. Reproduced with permission of Ref. [177] (Copyright American Chemical Society 2012). (**i**) Digital photograph of UFAs with diverse shapes; (**j**) A 100 cm^3^ UFA cylinder standing on a flower like dog’s tail; (**k**,**l**) Microscopically porous architecture of a UFA at different magnifications, showing CNT-coated graphene cell walls. Reproduced with permission of Ref. [155] (Copyright Wiley-VCH Verlag GmbH & Co. 2013).

Graphene-based aerogel is another kind of novel carbon aerogels, which was first prepared by Wang *et al.*, in 2009 [51]. GO solution was converted into the graphene aerogel by ultrasonic-induced gelation, drying and thermal reduction. Different from the physical crosslinking, Worsley *et al.* [172] reported the preparation of graphene aerogels crosslinked by chemical bonding through organic sol-gel chemistry. These graphene aerogels exhibit an improvement in bulk electrical conductivity (~1 × 10^2^ S·m^−1^), which is more than 2 orders of magnitude compared to that (~5 × 10^−1^ S·m^−1^) of graphene assemblies with physical crosslinks alone. The graphene aerogels also possess large surface area (584 m^2^·g^−1^) and pore volume (2.96 cm^3^·g^−1^), being viable candidates for use in energy storage, catalysis, and sensing applications. Following this pioneering work, Shi and coworkers reported the formation of a stable GO/PVA hybrid hydrogel due to the strong hydrogen bonding interactions between hydroxyl-rich PVA chains and oxygen-containing groups on GO sheets. Subsequent studies in the field confirmed that DNA, protein, synthetic polymers with cationic charges and hydrogen bonding acceptors, small quaternary ammonium salts and metal ions are efficient crosslinkers for GO gelation, which can regulate the delicate balance between electrostatic repulsion, hydrophobic interactions and hydrogen bondings of GO-based colloid systems [173,174,175,176]. In another report, an aqueous GO suspension with appropriate concentration was directly submitted to hydrothermal treatment to form reduced graphene oxide (rGO) hydrogel without any other reagents [50]. After drying, the resulting rGO aerogel is conductive (5 × 10^3^ S·cm^−1^), thermally stable (25–100 °C) and mechanically strong (with storage modulus of 470 ± 20 kPa) (Figure 8a–d).

Besides 3D foam-like structures, graphene aerogels can be tailored into different macroscopic architectures, such as one-dimensional graphene aerogel fibers. Gao and coworkers first prepared continuous neat GO aerogel fibers with unique “porous core-dense shell” structure from flowing GO liquid crystals with lamellar ordering by the combination of spinning technology and ice-templating strategy [177]. After chemical reduction and annealing process, the aligned porous graphene aerogel fibers possess high specific surface area and fine electrical conductivity. Interestingly, graphene aerogel fibers have fine flexibility and can be folded, and the bent fiber can be stretched to its original straight shape without any fracture (Figure 8e,f). Graphene sheets stacked densely to form a wrinkled shell surface, and interconnected to form a porous part which is surrounded by the dense shell as shown by the SEM images in Figure 8g,h. This work solved the widely expressed conflicts between high porosity (meaning lightweight and high surface area) and high performance in both strength and electrical conductivity, which could be helpful in the future design and fabrication of advanced aerogel materials. An ultra-flyweight aerogel (UFA) was fabricated by the same group through freeze-drying aqueous solutions of CNTs and giant graphene oxide (GGO) sheets, followed by chemical reduction of GGO into graphene with hydrazine vapor [155]. SEM images show the UFA exhibits an interconnected, porous 3D framework of randomly oriented, crinkly sheets with continuous macropores ranged from hundreds of nanometers to tens of micrometers (Figure 8k,l). The density of the UFA achieves 0.16 mg·cm^−3^, which is the lowest density of ultralight materials ever reported. In addition, compression experiments on the UFAs showed a nearly complete recovery after 50%–82% compression, indicating superelasticity and complete fatigue resistance under large cyclic strains. The desirable multifunctional attributes of UFAs such as outstanding temperature-invariant elasticity, ultralow density, excellent thermal stability, and good electrical conductivity would further widen the applications of carbon aerogels.

#### 3.2.3. Carbon-Based Hybrid Aerogels

The structures and properties of carbon aerogels can be easily tuned by incorporation of metal species into the carbon framework. Three main strategies have been used to introduce metal species into the carbon framework. The first consists of dissolving the metal precursor in the initial sol solution. The second involves the use of a monomer derivative containing an ion exchange moiety that can be polymerized using sol-gel techniques. The repeating unit of the organic polymer contains a binding site for metal ions, ensuring the uniform dispersion of the dopant. Finally, the third strategy is to deposit the metal precursor on the organic or carbon aerogel.

**Figure 9:**
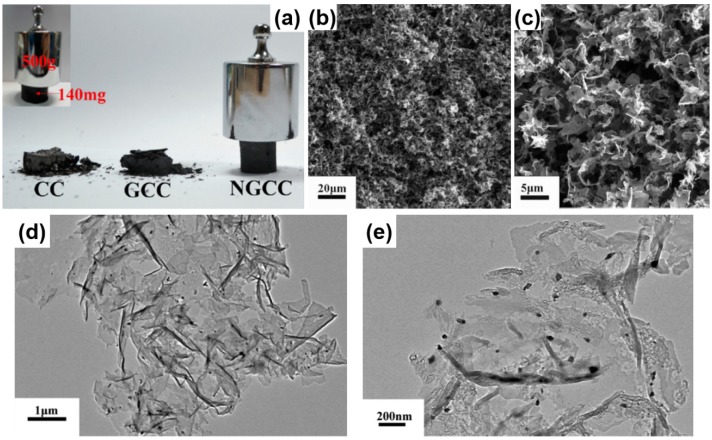
(**a**) Digital photographs of carbon cryogel (CC), graphene/carbon cryogel (GCC), and Ni-doped graphene/carbon cryogel (NGCC) with the same weight (90 mg) after loading with 200 g weight; (inset) 140 mg Ni-doped graphene/carbon cryogel monolith enduring a weight of 500 g. (**b**,**c**) SEM images and (**d**,**e**) TEM images of Ni-doped graphene/carbon cryogel. Reproduced with permission of Ref. [179] (Copyright American Chemical Society 2013).

Mo-doped carbon aerogels were obtained in the polycondensation reaction of aqueous resorcinol and formaldehyde by adding Mo-salt either into the initial mixture or the dried polymer aerogel by incipient wetting impregnation [178]. It was found that Mo-salt added during the polymerization process yielded a more compact gel structure with practically no mesoporosity. With post-impregnation, by contrast, mesopores with diameter of 3–15 nm were generated. Moreover, the content of Mo nanoparticle in the samples was also different. For the former one, Mo was lost during the solvent exchange process before supercritical drying (*i.e.*, Mo failed to bind chemically to the polymer matrix). The residual Mo congregated into bulk clusters of α-Mo_2_C with size of 25–60 nm. For post-impregnation, finely dispersed α-Mo_2_C and η-Mo_3_C_2_ crystals with the size of 8–20 nm formed on the surface of both carbon and Mo-based oxides. Ni-doped graphene/RF-based carbon cryogels were prepared using Ni^2+^ ions as catalysts for the gelation process to substitute the usually used alkaline carbonates [179]. The metal ions of Ni^2+^ effectively elevated the cross-linking between GO and RF skeletons, thus strengthening the whole cryogel. The as-formed 3D interconnected structures, which can be well-maintained after freeze-drying of the hydrogel precursor and subsequent carbonization under an inert atmosphere, exhibit good mechanical properties as shown in Figure 9. During the carbonization process, Ni^2+^ ions are converted into Ni nanoparticles and uniformly embedded in the interconnected structures. Therefore, the unique porosity within the interconnected structures endows the cryogels with good capability for extraction of oils and some organic solvents while the bulk form enables its recycling use.

**Figure 10:**
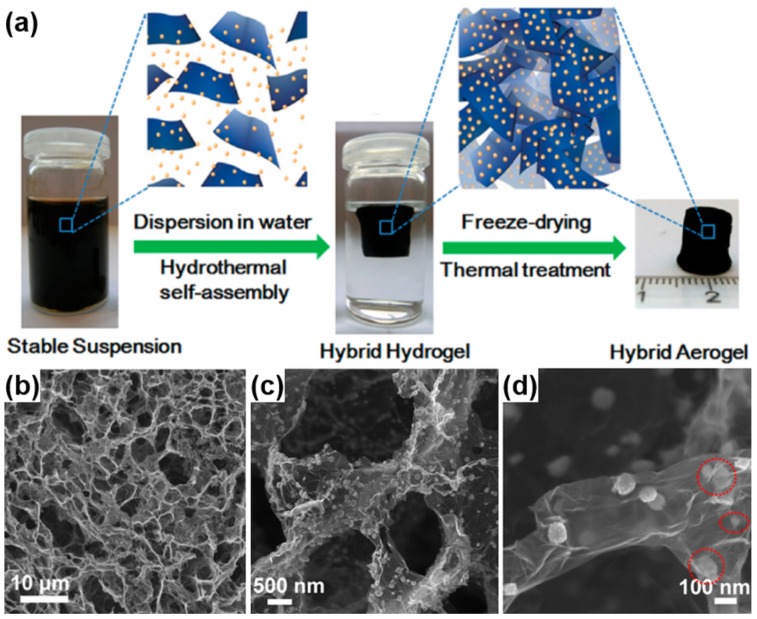
(**a**) Fabrication process for the 3D Fe_3_O_4_/N-doped graphene aerogel catalyst. (**b**–**d**) SEM images of Fe_3_O_4_/N-doped graphene aerogel revealing the 3D macroporous structure and uniform distribution of Fe_3_O_4_ nanoparticles in the graphene aerogels. Reproduced with permission of Ref. [62] (Copyright American Chemical Society 2012).

In addition, the high surface area and porosity of carbon aerogels make them efficient scaffolds for immobilizing inorganic nanoparticles to meet the demand for multi-functions. Various inorganic components such as metal oxides (Fe_3_O_4_, Fe_2_O_3_, MnO_2_, SnO_2_, WO_3_, ZnMn_2_O_4_, *etc.*), metal hydroxides (Ni(OH)_2_), and metal sulfides (MoS_2_, WS_2_) have been incorporated into carbon aerogels, and applied in catalysis, energy storage or water treatment. For example, WO_3_ decorated carbon aerogels were fabricated by carbonization of RF aerogel followed by a simple immersion-calcination process and used as electrode materials for supercapacitors [60]. WO_3_ in the carbon aerogels show a form of single crystalline nanoparticles of 15–40 nm in size. One order of magnitude improvement in specific capacitance was achieved, from 54 F·g^−1^ for WO_3_ nanoparticles to 700 F·g^−1^ for WO_3_/carbon aerogel composites. 3D N-doped graphene aerogel-supported Fe_3_O_4_ nanoparticles are reported as efficient cathode catalysts for oxygen reduction reactions. As shown in Figure 10, the graphene hybrids exhibit an interconnected macroporous framework of graphene sheets with uniform dispersion of Fe_3_O_4_ nanoparticles, exhibiting improved oxygen reduction performance [62]. Yin *et al.* [61] fabricated spinel ZnMn_2_O_4_/carbon aerogel composites with a 3D porous structure through a facile solution immersion chemical route. The hybrids show enormous interfacial surface area, connected 3D framework, abundant porosity and much higher capacity than that of pure spinel ZnMn_2_O_4_. These works further demonstrate the importance of 3D macropores and high specific surface area of the carbon aerogel support for improving the overall performance of the hybrid materials.

## 4. Applications of Polymer/Carbon-Based Aerogels

### 4.1. Energy Storage

The development of electrochemical systems with high power and energy densities for sustainable energy storage has been considered as one of the critical solutions to resolve global concerns of fossil fuel depletion and environmental deterioration. Carbon aerogels with 3D porous architectures are excellent candidates for high performance electrode materials due to their high specific surface area and developed porosity, which provide continuous channels to facilitate ion transport by shortening diffusion pathways and ensuring excellent electrical contact.

The pore size distribution of carbon aerogels strongly affects the specific surface area and subsequently the electrochemical behavior. It has been proved that carbon aerogels with a pore diameter in the range from 3 to 13 nm exhibit the best voltammetry characteristics and the highest capacitance values (70–150 F·g^−1^). The carbon aerogels obtained at temperatures over 900 °C show an aggravation of specific capacitance, whereas functionalization of the carbon surface by heating at 500 °C in air environment could cause an improvement of the capacitance through the pseudocapacitance effects [22]. Moreover, Hall *et al.* [180] studied the effect of pore size of activated carbon aerogels on the capacitance performance in ionic liquid electrolytes. The best capacitance performance was obtained for the activated carbon aerogel with average pore diameter of 3.5 nm, whereas the optimum rate performance was obtained for the activated carbon aerogel with average pore diameter of 6 nm. When combined in an electrochemical capacitor with ionic liquid electrolyte EMImBF_4_, a specific capacitance of 210 F·g^−1^ was obtained for activated carbon aerogel with average pore diameter of 3.5 nm at an operating voltage of 3 V. On the other hand, the activated carbon aerogel with average pore diameter of 6 nm allowed for maximum capacitance retention of approximately 70% at 64 mA·cm^−2^.

However, the specific capacitance of pure carbon aerogels is very limited due to its intrinsic electrical double layer behavior. By activating the carbon aerogels in different gases or chemical substance, the porous structure and surface area of the concerned carbon aerogels can be improved. It was reported that the porous structure and electrochemical performance of the carbon aerogels were significantly affected by chemical activation of KOH [159]. Macro and mesoporous carbon was first prepared by carbonizing the freeze-dried bagasse aerogel, and microporous structure was consequently created on the walls of the mesoporous carbon by chemical activation. The KOH-activated carbon aerogels have a typical hierarchical porous structure. The first level is large macropores, which are larger than 50 nm in diameter. The second level is the mesopores in the channel wall of the large pores, which is composed of several connections of carbon nanoparticles and is about 2–50 nm in diameter. And the third level is micropores in the carbon nanoparticles, which is composed of graphite-like structures and is smaller than 2 nm. As a result, the KOH-activated carbon aerogels show a high specific capacitance of 268.4 F·g^−1^ at a scan rate of 2 mV·s^−1^, which is about four times higher than that of the unactivated one. In addition, the capacitance of carbon aerogels could be improved by heteroatom doping. For example, N-doped carbon aerogels were synthesized by using watermelon as the starting materials and polypyrrole as the N-source via a hydrothermal method [157]. The obtained aerogels exhibit excellent electrochemical performance, in particular, an outstanding specific capacitance of 281.0 F·g^−1^ can be achieved for the sample calcined at 600 °C. The improvement of electrochemical performance is attributed to the combinatorial effect of the appropriate N content and porous structure.

Another method is to incorporate electrically active materials such as metal oxides or conductive polymers into carbon aerogels to improve their electrochemical performance. MnO_2_ loaded carbonaceous aerogel (MnO_2_@CA) composites were also synthesized via the hydrothermal carbonaceous process [181]. The obtained MnO_2_@CA displays the specific capacitance of 123.5 F·g^−1^ at 5 mV·s^−1^. The enhanced supercapacitance of MnO_2_@CA nanocomposites could be ascribed to both electrochemical contributions of the loaded MnO_2_ nanoparticles and the porous structure of 3D carbonaceous aerogels. LiFePO_4_/carbon hybrid aerogels were prepared by mixing precursor solution with carbon aerogel via a simple solution impregnation method and used as cathode for lithium ion batteries [182]. LiFePO_4_ nanowires forming on the ektexine of carbon aerogel intertwined with LiFePO_4_/CA particles and formed a special web structure, which can improve the initial discharge capacity up to 139.3 mAh·g^−1^. The added carbon aerogel induces the growth LiFePO_4_ nanowire and increases the electronic conductivity while the formation of 3D nano-network increases the contact area between active materials and electrolyte. Thus, both the specific capacity and the cycle stability of LiFePO_4_ at high discharge rate can be significantly improved.

**Figure 11:**
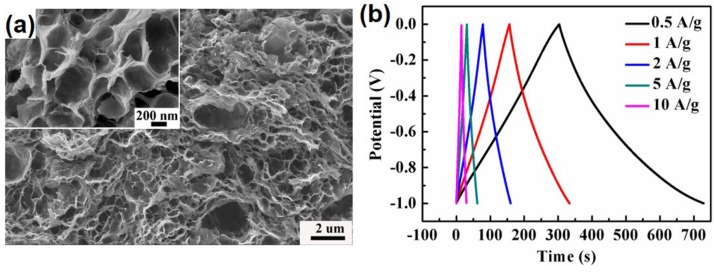
(**a**) SEM images and (**b**) galvanostatic charge–discharge curves of graphene/carbon aerogels prepared from carbonization of graphene crosslinked PI aerogels. Reproduced with permission of Ref. [185] (Copyright The Royal Society of Chemistry 2015).

Recently, graphene aerogels draw great attention owing to their unique properties and excellent performance in energy-related fields. As an electrode material for supercapacitors, the obtained graphene foam has electrical conductivity of 5 × 10^−3^ S·cm^−1^, and exhibit a specific capacitance of 175 F·g^−1^ in an aqueous electrolyte [50]. Subsequently, in order to obtain higher conductivity of graphene foam, the authors used a reducing agent (hydrazine or hydroiodic acid) for further reduction of the foam [183]. In this case, the chemically reduced graphene foam possessed high conductivity of 1.3–3.2 S·m^−1^, high specific capacitance of 220 F·g^−1^ and higher rate capability compared to their previous work. Following this pioneering work, graphene incorporated or crosslinked carbon aerogels were extensively studied. Worsley *et al.* [172] demonstrated that GO aqueous suspension can be cross-linked with RF using sodium carbonate as catalyst by sol-gel chemistry, followed by supercritical CO_2_ drying and pyrolysis at 1050 °C under nitrogen atmosphere. The as-prepared macroscopic 3D graphene foam exhibited excellent electrical conductivity (100 S·m^−1^), high surface area (584 m^2^·g^−1^) and large pore volume (2.96 cm^3^·g^−1^). Subsequently, the porous structure of graphene foam was adjusted by increasing the RF content, and as a result, the obtained foam had a high specific surface area of 1200 m^2^·g^−1^ and a large pore volume of 6 cm^3^·g^−1^, and maintained its high conductivity [184]. Graphene-carbon aerogel composites with tailored pore structure and morphology were synthesized by a facile yet effective method with ionic liquids as templates [92]. They indicated that the incorporation of graphene can increase the specific capacity of carbon aerogels from 140 F·g^−1^ to 230 F·g^−1^ at a current density of 0.1 F·g^−1^ without using other activation agents. Furthermore, the electrode materials prepared from graphene-carbon aerogels show high cyclic stability even after 5000 cycles. Graphene/carbon aerogels with multimodal pores were reported via carbonization of graphene crosslinked PI aerogels [185]. Compared to most carbon aerogels prepare previously, this preparation process is facilitated by the exclusion of harmful formaldehyde. Moreover, as a powerful crosslinking agent, graphene is beneficial for acceleration of the gelation process, improvement of the porous structures inside carbon aerogels and enhancement of the specific surface area and electrical conductivity of carbon aerogels. SEM observation (Figure 11) shows the multimodal pores and 3D nano-network of carbon aerogels, which provide high electroactive regions and short diffusion lengths for both charge and ion transport. Owing to the incorporation of graphene, the as-prepared carbon aerogels possess high specific surface area up to 998.7 m^2^·g^−1^ and specific capacitance up to 178.1 F·g^−1^ at a current density of 1 A·g^−1^, which is much higher than that of pure carbon aerogels (193.6 m^2^·g^−1^ and 104.2 F·g^−1^). This work provides a new and facile method for fabricating high performance carbon aerogels with hierarchical structures and extends the potential applications of polyimide.

Overall, electrodes based on carbon aerogels possess good electrochemical performance, high reversibility and high specific capacitance. Moreover, the utilization efficiency of carbon aerogels is promoted due to their excellent electronic conductivity and extensive mesoporous network of carbon aerogels. Therefore, carbon aerogels with high surface area and electrical conductivity are considered as promising electrode materials for applications in high-performance supercapacitors.

### 4.2. Absorption

It is urgent to develop an economic and feasible strategy to solve the environmental and ecological problems arising from the increasing pollution from crude oils, petroleum products and toxic organic solvents. Due to their high porosity and high hydrophobicity, carbon aerogels have been extensively investigated as candidates for removal of pollutants and separation of oil from water [156,160,186,187]. As listed in Table 1, carbon aerogels usually exhibit much higher sorption capacity (up to 900 g·g^−1^) compared with other solid materials such as polymers, graphite or activated carbon, which only have sorption capacities less than 100 g·g^−1^.

**Table 1:** Comparison of various aerogel materials for adsorption.

Sorbent Materials	Absorbed Substances	Sorption Capacity (g·g^−1^)	Ref.
Graphene/PVDF aerogels	Oils and organic solvents	20–70	[32]
PVA/CNF aerogels	Oils and organic solvents	44–96	[33]
CNF aerogels	Water, oils and organic solvents	139–375	[111]
Ultra-flyweight aerogels	Oils and organic solvents	215–913	[155]
Winter melon carbon aerogels	Oils and organic solvents	16–50	[156]
Carbon microbelt aerogels	Oils and organic solvents	50–188	[160]
Ni-doped graphene/carbon cryogels	Oils and organic solvents	22.2–23.2	[179]
Twisted carbon fiber aerogels	Oils and organic solvents	50–192	[186]
Vegetable fiber	Crude oil	1–100	[188]
Nanowire membrane	Oils and some organic solvents	4–20	[189]
Wool-based nonwoven	Diesel, crude oil, SN 150	9–15	[190]
Polymers	Oils and organic solvents	5–25	[191]
Exfoliated graphite	Heavy oil	60–90	[192]
Activated carbons	Benzene, toluene	<1	[193]
Carbon nanotube sponges	Oils and organic solvents	80–180	[194]
Graphene/CNT foam	Compressor oil, organic solvents	80–140	[195]
CNT sponge doped with boron	Oils and organic solvents	25–125	[196]
Graphene-based sponges	Oils and organic solvents	60–160	[197]
Reduced graphite oxide foam	Cyclohexane, chlorobenzene, toluene, petroleum, motor oil	5–40	[198]
Nitrogen doped graphene foam	Oils and organic solvents	200–600	[199]
Carbonaceous nanofiber aerogels	Oils and organic solvents	40–115	[200]

**Figure 12:**
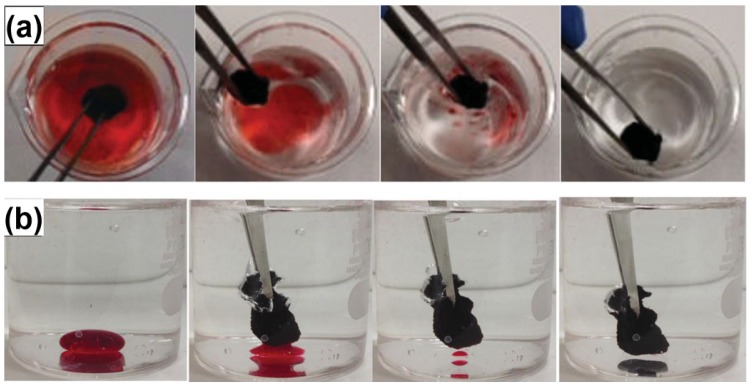
(**a**) Photographs showing the sorption process of heptane by using a TCF aerogel taken at intervals of 10 s. Heptane stained with Sudan red 5B floating on water was completely absorbed within 40 s. (**b**) Photographs showing the sorption process of chloroform by using a TCF aerogel. Chloroform stained with Sudan red 5B at the bottom of water was completely absorbed within 5 s. Reproduced with permission of Ref. [186] (Copyright Wiley-VCH Verlag GmbH & Co. 2014).

The hydrophobicity of carbon aerogels makes it an ideal candidate for removal of pollutants and separation of oil and water, because its hydrophobic surface can selectively and effectively adsorb oily target compounds mixed with water. Lightweight, hydrophobic and porous aerogels made from twisted carbon fibers (TCF) are produced by Bi and coworkers via a facile route by using cotton fibers as raw materials [186]. Notably, the TCF aerogel can adsorb a wide range of organic solvents and oils with a maximum sorption capacity up to 192 times of the weight of the pristine TCF aerogel (Figure 12). Moreover, the TCF aerogel also exhibits the excellent recyclability, and maintains a high sorption capacity even after five cycles through distillation, burning or squeezing. Thereafter, the same group produced aerogels made of carbon microbelts (CMBs) by using waste paper as the raw material [160]. The CMB aerogel can adsorb a wide range of organic solvents and oils with a maximum sorption capacity up to 188 times of the weight of the pristine CMB aerogel. Most importantly, the abundant source and simple preparation method make the CMB aerogel cost-effective for possible industrial applications, such as barrier separation and water purification.

**Figure 13:**
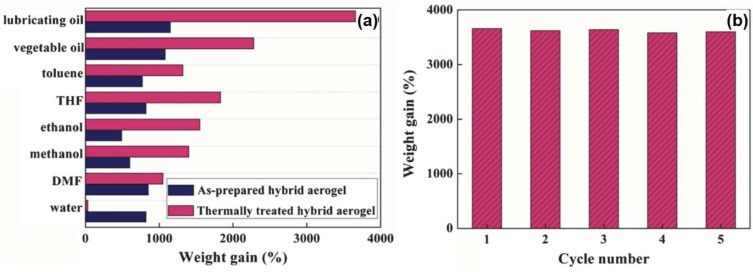
(**a**) The adsorption capacities of the MWNT-carbon hybrid aerogels for a selection of several common solvents. (**b**) Cyclic performance of the thermally treated MWNT-carbon hybrid aerogels for oil adsorption in five cycles. Reproduced with permission of Ref. [202] (Copyright The Royal Society of Chemistry 2013).

Nitrogen-rich carbon (NRC) aerogels with highly hydrophobic surfaces were reported by Yang and coworkers [201]. The NRC aerogels can be used as oil-sorbent materials to adsorb oils or organic solvents in oil-spilled accidents by an adsorption/distillation or adsorption/combustion ways. The NRC aerogels can maintain 100% of their initial adsorption capacities after 100 adsorption/distillation cycles and 61.2% after 100 adsorption/combustion cycles, which thus has the highest recyclability of the reported carbon aerogels. Gao *et al.* [179] prepared Ni-doped graphene/carbon cryogels and further used as versatile sorbents. The cryogel monoliths exhibit ultrafast absorption rate and high uptake capacity for oils and organic solvents, such as trichloromethane (close to 50 g·g^−1^), with a good recyclability. MWNT-carbon hybrid aerogels with 3D interconnected frameworks and low density of about 0.02 g·cm^−3^ were via a one-pot hydrothermal carbonization process [202]. The uptake capacity of a thermally treated MWNT-carbon hybrid aerogel is up to about 37 times of its weight for various lubricating oils, and up to about 23 times for vegetable oil (as shown in Figure 13). It is worth to mention that the thermally treated MWNT-carbon hybrid aerogel shows a good recyclability to these solvents, as about 98% of oil removal efficiency can still be retained even after five cycles of adsorption–desorption process by using hexane as an extractant.

Except oils and organic solvents, carbon aerogels can also be applied to remove heavy metal ions from water. It was found that the removal of heavy metal ions such as Cd(II), Pb(II), Hg(II), Cu(II), Ni(II), Mn(II) and Zn(II) by carbon aerogel was influenced by concentration, pH, contact time, adsorbent dose and temperature [203]. Superhydrophobic and superoleophilic PVA/CNF hybrid aerogels were reported after being treated with methyltrichlorosilane via a simple thermal chemical vapor deposition process [33]. The silane-treated PVA/CNF aerogels not only exhibited excellent absorption performance for various types of oils (e.g., crude oil) or organic solvents, but also showed a remarkable scavenging capability for several types of heavy metal ions. The scavenging capacities of the PVA/CNF aerogels for Hg^2+^, Pb^2+^, Cu^2+^, and Ag^+^ were 157.5 mg·g^−1^, 110.6 mg·g^−1^, 151.3 mg·g^−1^, and 114.3 mg·g^−1^, respectively, which are greatly improved compared to pure PVA aerogels. It is believed that metal ion scavenging in aqueous solutions by porous materials such as aerogels is mainly driven by electrostatic interaction and complexation between the metal ions and the carboxyl groups present in the porous materials. The remarkable scavenging heavy metal ions capability exhibited by PVA/CNF aerogels makes them versatile absorbents for various potential applications including water purification.

### 4.3. Adsorption

Gas purification represents a major challenge in a time of concerns for growing air pollution based on emission of gases (especially toxic and greenhouse gases) from various industrial sources. In particular, the subject of carbon dioxide (CO_2_) capture, utilization, and storage has received widespread attention because of the interest in reducing the amount of released CO_2_ as a greenhouse gas. The amount of CO_2_ present in the atmosphere contributes to 60% of global warming effects [204]. Hence, it is particularly urgent to prevent the release of carbon dioxide and lower their concentration in the atmosphere. Many CO_2_ capture technologies, such as absorption, cryogenic, adsorption, and membranes, have been investigated. Among them, adsorption on solid adsorbents is considered to be one of the most promising approaches to deal with environment related problems. There is intense focus on developing simple and cheap synthesis methodologies that deliver highly porous materials with appropriate properties for use as CO_2_ capture. In this regard, aerogels are attractive due to their high porosity, versatility, low bulk density, and high internal surface area [205]. Therefore, recent investigations have focused on using aerogels as new CO_2_ capture materials. Masika developed a carbon aerogel with a high surface area of 1100 m^2^·g^−1^, and the CO_2_ adsorption capacity obtained in pure CO_2_ at 25 °C and 1 bar was only 2.2 mmol·g^−1^ [206]. Chemical activation of the carbon aerogel with KOH generates activated carbon aerogels with surface area between 915 and 1980 m^2^·g^−1^ and pore volume of up to 2.03 cm^3^·g^−1^ with the extent of the increase in textural properties. The activated carbon aerogels show a micropore size distribution centred at ca. 8 and 13 Å, with a large increase in pore volume and proportion of microporosity. As a result, the activated carbon aerogels exhibit attractive CO_2_ uptake of between 2.7 and 3.0 mmol·g^−1^ at 25 °C and 1 bar under flowing pure CO_2_ conditions [207]. 3D porous carbon through steam activation of graphene aerogel was also reported for CO_2_ uptake [208]. The steam activated graphene aerogel exhibits a 3D network structure with interconnected pores and possesses a high BET specific surface area (830–1230 m^2^·g^−1^) and a large pore volume (2.2–3.6 cm^3^·g^−1^). CO_2_ adsorption capacity (10.8 wt %) for steam activated graphene aerogel is much higher than that (4.1 wt %) of graphene aerogel prepared without steam activation, indicating that steam activation is of great importance to the enhancement of CO_2_ adsorption. A hybrid aerogel material composed of chitosan and graphene oxide was developed by Alhwaige and coworkers [209]. It is demonstrated that dispersing GO into chitosan matrix, in the form of aerogel, leads to increased surface area with subsequent increase in CO_2_ sorption. The amount of CO_2_ adsorbed at 25 °C increases from 1.92 to 4.15 mol·kg^−1^ with the addition of 20 wt % GO. Adsorption–desorption cycles exhibit the stability of the hybrid aerogels during prolonged cyclic operations, suggesting excellent potential for CO_2_ capture technology.

### 4.4. Thermal Insulations

The energy needed to maintain a pleasant interior atmosphere accounts for more than 10% of the gross global energy consumption. Thermal insulation plays a major role in controlling the energy efficiency of buildings or aerospace and it is needed to substantially reduce thermal conductivity (λ) values below those of currently used insulation materials such as expanded polystyrene (EPS; λ = 30–40 mW·m^−1^·K^−1^), polyurethane (λ = 20–30 mW·m^−1^·K^−1^), glass fiber (λ = 33–44 mW·m^−1^·K^−1^) and mineral wool (λ = 30–40 mW·m^−1^·K^−1^). In fact, significantly reducing λbelow the value for air (25 mW·m^−1^·K^−1^) is desirable to minimize the required space and materials without severely compromising the architectural design. Approaches to obtain super-insulating materials include the replacement of air with another gas or vacuum or by reducing the pore size below the mean free path of air [210,211]. However, it is challenging to maintain a special gas or vacuum inside insulating panels for an extended time, which can result in a loss of the initially low thermal conductivity with time. Therefore, nanoporous aerogels are ideal candidates for both ambient and high temperature insulation applications owing to their unique nanostructure, high porosity and lightweight. Generally, heat is transferred by radiation, conduction or convection. Due to the small pore sizes of aerogels (1–100 nm), the convective and gas conduction pathways are virtually eliminated. Hence, what remains is primarily radiative heat transfer and conduction through the aerogel network.

Silica aerogels can be used under ambient conditions and still maintain a low λ of ~17–21 mW·m^−1^·K^−1^, but they are brittle and difficult to prepare in large sizes. Biopolymer-based materials such as wood chips, recycled paper and cork were extensively used for thermal insulation before the introduction of fossil-fuel-based foams, but their insulating performance is relatively poor with thermal conductivities of 40–50 mW·m^−1^·K^−1^. However, with the emergence of nanocellulose extracted from wood and other sources, there are now possibilities for the nanoscale engineering of renewable materials to generate more efficient thermal insulators. Cellulose aerogels were fabricated from nanocellulose fibers with different morphologies and surface properties derived from biomass resources [212]. Though the structures of aerogels are different, all cellulose aerogels show a thermal conductivity lower than 0.016 W·m^−1^·K^−1^, which can be used as thermal insulation materials. Pectin aerogels were prepared via dissolution-gelation-coagulation and subsequent drying with supercritical CO_2_ [87]. The resulting pectin aerogels have a thermal conductivity ranging from 0.016 to 0.022 W·m^−1^·K^−1^, which is below that of air in ambient conditions, making them new thermal super-insulating fully biomass-based materials. However, it remains a big challenge for biopolymer-based insulation materials due to their poor resistance to fire and their moisture sensitivity. Recent work has shown that nanomaterials such as clays and graphenoids can provide organic polymer-based nanocomposites with good fire retardancy and excellent mechanical properties. Thus, graphene oxide and sepiolite nanorods were incorporated into the suspensions of cellulose nanofibers to prepare cellulose-based aerogels [35]. The aerogels are super-insulating, showing a thermal conductivity of 15 mW·m^−1^·K^−1^ and excellent combustion resistance, which is about half of that of expanded polystyrene. At 30 °C and 85% relative humidity, the foams retained more than half of their initial strength. The results show that nanoscale engineering is a promising strategy for producing foams with excellent properties by using cellulose and other renewable nanosized fibrous materials. Similarly, nanoporous hybrid aerogels based on cellulose nanofibers and nanozeolite particles were fabricated to achieve lower thermal conductivity of these materials. Thermal conductivity value as low as 18 mW·m^−1^·K^−1^ was obtained that confirms the superinsulation ability of these new fibrous aerogels. Synergism on the thermal conductivity properties was shown by adjunction of nanozeolites to cellulose microfibrils by reaching pore size lower than 100 nm that significantly reduces the thermal conductivity of the hybrid aerogels [213].

PI aerogels are ideal candidate for thermal insulation materials, especially in aerospace applications when much higher use temperatures are needed, such as insulation for launch vehicles or for planetary entry, descent, and landing systems. Aramid aerogels prepared by efficient reaction of carboxylic acids and isocyanates show a low thermal conductivity of 0.028 W·m^−1^·K^−1^ [214]. Based on this result, the authors further synthesized PI aerogels by ring-opening metathesis polymerization of a norbornene end-capped diimide [146]. The new materials combine facile one-step synthesis with heat resistance up to 200 °C, high mechanical compressive strength and specific energy absorption (168 MPa and 50 J·g^−1^, respectively, at 0.39 g·cm^−3^ and 88% ultimate strain), low speed of sound (351 m·s^−1^ at 0.39 g·cm^−3^) and styrofoam-like thermal conductivity (0.031 W·m^−1^·K^−1^ at 25 °C). Therefore, they are reasonable multifunctional candidate materials for further exploration as thermal/acoustic insulation at elevated temperatures. Apart from thermal conductivity, decomposition temperature of aerogels as well as its dependency on external conditions is usually discussed for different classes of thermal insulation aerogels. It is reported that the onset decomposition temperature of crosslinked aromatic PI aerogels can reach up to 525 °C to 625 °C [143,144,146]. Consequently, PI aerogels can be used for aircraft engine applications because of their high temperature stability, low density and low thermal conductivity.

In summary, polymer aerogels have a similar thermal conductivity with that of silica aerogels, which is about 0.016 W·m^−1^·K^−1^, but much stiffer and stronger than silica aerogels. Therefore, polymer aerogels have potential applications as thermal insulation materials with improved thermal stability and mechanical properties, which can meet the requirement in aerospace.

### 4.5. Flame Retardant Materials

Even though polymer aerogels are excellent thermal insulation materials, their applications in flame retardant materials are limited by their poor resistance. Therefore, modifications are required to decrease their flammability through the addition of flame retardant compounds. Environmental regulation has restricted the use of some halogenated flame retardant additives, initiating a search for alternative flame retardant additives. Recent work has shown that nanoparticle fillers, such as clays, graphene and CNTs, are highly attractive for this purpose, since they can simultaneously improve both the mechanical properties and flammability of the polymer aerogels.

Preparation of polymer/clay aerogel materials through a freeze drying process was reported and intensively studied by Schiraldi and coworkers [17,36,37,215,216]. They fabricated low flammable aerogels based on renewable ammonium alginate and MMT clay through a simple, environmentally-friendly freeze-drying process [215]. Alginate is a polyelectrolyte derived from seaweed that is considered to be biocompatible, non-toxic, non-immunogenic and biodegradable, and it has been proved to be an inherently flame-retardant material with limited oxygen index value of 48.0, compared with 20.0 for viscose fiber. During cone calorimetry tests, the peak heat release rate and effective heat of combustion of alginate fiber were 4.99 kW·m^−2^ and 0.46 MJ·kg^−1^, respectively, compared with 168.75 kW·m^−2^ and 12.06 MJ·kg^−1^ for viscose fiber. With addition of clay, the flammability of alginate/clay aerogels was further reduced. No open fire was observed during testing of these aerogels, and 53 wt % of sample weight remained with the original sample shape remaining intact. With a high concentration of clay (50 wt % of the solid), the silicate layers with high aspect ratio served as heat and mass transport barriers, which would largely decrease the burning rate. Furthermore, ceramic transformation of high concentration of clay particles helps to form stable structure of sample, and inhibit the flame spread. Thereafter, a series of PVA hybrid aerogels were prepared by using different nanofillers, e.g., nanoscale silica, halloysite, MMT and laponite through the above mentioned freeze-drying method [36]. The aerogels with layered structures have lower flammability than those non-layered aerogels and the heat release values of aerogels are much lower than those for commercial expanded polystyrene foam. Based on these results, they further prepared PVA/MMT aerogels followed by radiation crosslinking to increase the mechanical properties of aerogels [37]. The cross-linked PVA/MMT aerogels possess very low flammability and high compressive modulus, especially at the absorbed dose of 30 kGy. For the mechanism of flammability, they indicated that with increasing clay content, the heat and mass transport barriers are developed, simultaneously low levels of thermal conductivity are maintained during burning. Furthermore, a facile preparation of PVA/MMT hybrid aerogels was introduced by using water as solvent and divinylsulfone as crosslinking agent [17]. The compressive modulus of PVA/MMT aerogels can increase 29-fold upon crosslinking, which show a low flammability and a great potential for applications in flame retarded materials.

**Figure 14:**
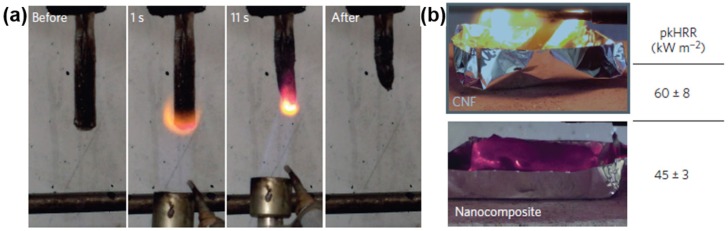
(**a**) Vertical burning test of a nanocomposite foam containing 77% CNF, 10% GO, 10% SEP and 3% BA (in wt %). The panel shows the foam before the test, after 11 s of application of a methane flame, and the foam after the test, showing high fire retardancy. (**b**) Photographs of CNF and CNF-GO-BA-SEP nanocomposite foams during the cone calorimetry test together with the corresponding peak of heat release rates (pkHRR). The test reveals high combustion resistance for the nanocomposite foam at the limit of non-ignitability, while CNF foams are entirely combusted. Reproduced with permission of Ref. [35] (Copyright Nature Publishing Group 2014).

High-performance flame retardant materials from renewable resources are desirable for improving the energy efficiency of buildings. Traditional fossil-fuel-derived flame retardant materials such as expanded polystyrene and polyurethane have thermal conductivities that are too high for retrofitting or for building new, surface-efficient passive houses. Therefore, lightweight, highly porous cellulose nanofibers (CNF)-GO-sepiolite nanorods (SEP) foams were produced by freeze-casting and exhibit excellent combustion resistance [35]. Freeze-casting offers a versatile approach to producing highly anisotropic porous materials, in which ice crystals grow with the temperature gradient and eventually produce a frozen material consisting of anisotropic ice crystals surrounded by the walls formed by the dispersed nanoparticles. As a consequence, the anisotropic nanocomposite foams are mechanically stiff in the freezing direction and able to sustain a considerable load. Vertical burning tests (UL 94) show that nanocellulose-based composite foams with an optimized addition of GO (10 wt %), SEP (10 wt %) and boric acid (BA) (3 wt %) display excellent fire retardancy, where the flame does not self-propagate (as shown in Figure 14). For the optimal foam composition, the limiting oxygen index, which gives the oxygen concentration (in %) needed to keep a material burning, is as high as 34, that is 60% higher than the O_2_ level in air (21%). In addition, it was found that CNF-GO-BA-SEP nanocomposite foams lie on the border between only smoldering and ignition when exposed to a defined heat flux of 35 kW·m^−2^, in contrast even to halogenated polyurethane foams or layer-by-layer modified polymer foams, which always ignite under similar conditions. The results provide substantial motivation to continue the development of high-performance thermal insulating materials based on renewable or widely abundant resources for the improvement of energy efficiency and reduction of the environmental impact of buildings.

PI aerogels not only have the typical characteristics of ordinary polymer aerogels but also show good heat resistance. As a result, PI could be used to strengthen clay-based aerogels while retaining the good thermal stability of these materials. However, almost all PIs cannot be dissolved in water, which restricts the direct introduction of PI into clay aerogels. In order to solve this problem, Wu *et al.* [40] prepared PAA ammonium salt to be used as a water-soluble precursor to produce PI/MMT hybrid aerogels. The resulted PI/MMT aerogels show a “layer-strut” bracing structure, which can improve the mechanical performance and heat resistance of aerogels. Besides, the onset decomposition temperature of the aerogels reached up to 410 °C. The combination of PI and MMT raises the possibility that these modified hybrid aerogels could be used as high-temperature insulation and flame-retarded materials.

**Figure 15:**
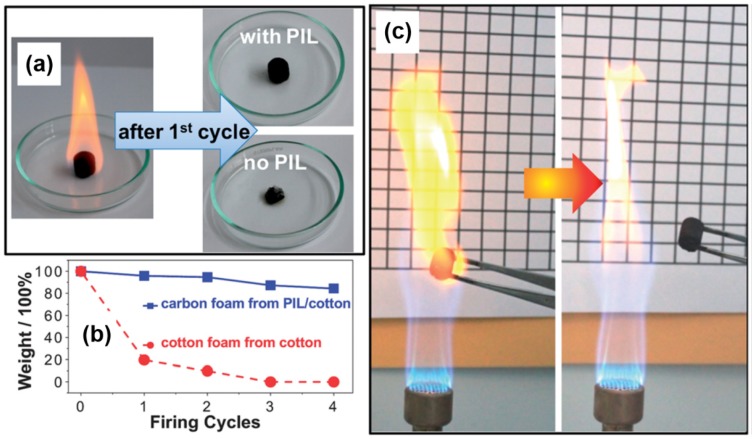
(**a**) Photographs of the 1st firing cycle test of the carbon foams (absorb 500 wt % of ethanol and burn in air): (**Left**) a burning ethanol-wetted foam and (**Right**) foams after the 1st firing cycle test. (**b**) Plot of the carbon foam mass *vs.* the firing cycle. (**c**) Photographs illustrating the fire-retardancy of a carbonaceous foam by repeatedly firing the sample using a butane/propane gas burner. Reproduced with permission of Ref. [217] (Copyright the Royal Society of Chemistry 2015).

In addition to polymer aerogels, carbon aerogels with hydrophobic surfaces and graphite skeletons also exhibit excellent fire resistant properties. Nitrogen-rich carbon (NRC) aerogels from alkaline lignin exhibit highly hydrophobic surfaces and excellent fire resistant property, making the NRC aerogels can be readily cleaned for reuse by direct combustion in air [201]. The excellent fire resistant properties are attributed to their graphite skeletons generated during the pyrolysis process. In addition, heteroatom doping can also attribute to the enhancement of fire resistant performance. Sugar-based molecules and polysaccharide biomass can be turned into porous functional carbonaceous products at comparably low temperatures of 400 °C under a nitrogen atmosphere in the presence of an ionic liquid (IL) or a poly(ionic liquid) (PIL) [217]. The IL and PIL act as “activation agents” with own structural contribution, and effectively promote the conversion and pore generation in the biomaterials even at a rather low doping ratio (7 wt %). Moreover, the nitrogen atoms incorporated in the final products from the IL/PIL precursors further improve the oxidation stability in the fire-retardant tests. The fire resistance of carbon aerogels from cotton with or without PIL were tested by wetting the foam with 500 wt % of ethanol and burning it in air (Figure 15a). As observed, the carbon aerogels derived from pure cotton already collapsed after the 1st cycle, while the PIL/cotton carbon remained intact. The mass *vs.* firing cycle plot (Figure 15b) indicates that the 1st firing cycle burnt away 80.3 wt % of the cotton-based carbon product while only 4.1 wt % of mass loss occurred for the cotton/PIL carbon aerogel. Within three cycles, only the cotton/PIL carbon aerogel preserved its shape and 85 wt % of its mass. Under even harsh conditions, for examples, the aerogel maintained its original shape and size without quenching fire when repeatedly exposed to the flame of a butane/propane gas burner (Figure 15c). This illustrates that the material is even robust against co-firing, a really exceptional property for high surface area carbons.

### 4.6. Catalyst Carriers

In order to improve the performance (general physicochemical characteristics as well as reactivity for catalysis) of catalysts, the most important characteristics are high surface area and phase stability of catalyst nanoparticles. Therefore, carbon aerogels with high conductivity, high mesoporosity and environmental stability are promising materials for application in catalysis [94].

The incorporation of metal or metal oxide species into the carbon framework can modify the structure, conductivity and catalytic activity of the carbon aerogel. Different metal-containing carbon aerogels have already been prepared and characterized, including Ce- and Zr-doped carbon aerogels and Cr-, Mo-, W-, Fe-, Ru-, Co-, Ni-, Pd-, Pt-, Cu- and Ag-containing carbon aerogels. The catalytic behavior of these metal-doped carbon aerogels with good activity and selectivity have been tested in a few reactions, including the isomerization of 1-butene, toluene combustion, synthesis of methyl-tert-butyl ether, the formation of nanofilaments from CO decomposition and cathode catalysts in proton exchange membrane fuel cell. For example, Mo-doped carbon aerogels were prepared in the polycondensation reaction of aqueous resorcinol and formaldehyde by adding Mo-salt at two different stages of synthesis [178]. The results show that the catalytic activity of carbon aerogels toward acetic acid hydroconversion reaction is enhanced by molybdenum. The more open pore structure, higher concentration and finer Mo distribution as well as its chemical form may all contribute to the greater conversion and higher value products obtained with the post-impregnated sample. γ-Fe_2_O_3_/α-Fe_2_O_3_/carbon aerogel is a magnetic photocatalyst, which shows strong Rhodamine B dye adsorption ability [218]. The removal capacity of RhB dyes of the as-prepared mesoporous aerogels is increased from 83.5% to 91% under visible light irradiation. The work shows that the as-prepared trifunctional mesoporous hybrid structures have great potential applications in wastewater treatments.

### 4.7. Hydrogen Storage

With the aggravation of the energy crisis, hydrogen energy has recently attracted increasing attentions. However, the main worldwide issue is to explore economic, safe, practical and convenient methods for hydrogen storage. The porous adsorbents for hydrogen storage are basically divided into four categories, *i.e.*, carbon-based porous materials, non-carbon nanotube materials, minerals and metal-organic porous materials. The basic requirements of hydrogen storage materials for porous solid adsorption are large surface area and high porosity. Therefore, carbon aerogels with large surface area, high porosity and structure stability have been studied for their potential applications in hydrogen storage [219]. However, the research of carbon aerogels as a hydrogen storage material is still in its initial stages. RF-based carbon aerogels are often used as the scaffold to store hydrogen. It is reported that carbon aerogels or aerogels catalyzed by acetic acid and activated by CO_2_ show a higher hydrogen uptake of 4.65 wt % at 77 K and 3.9 MPa than that of uncatalyzed or unactivited aerogels.

Solid-state hydrogen storage materials such as MgH_2_, LiBH_4_ and NaAlH_4_ offer high hydrogen storage capacities, which are required for a compact and efficient hydrogen storage system. However, until now, solid-state hydrogen storage has suffered from difficulties such as combination of high storage capacities in light element hydrides with hydrogen release. One approach is confinement of metal hydrides in inert mesoporous scaffolds with pore sizes in the range of 2–50 nm [220,221,222,223,224]. Therefore, carbon aerogels with nano-pore sizes are the right choice. Recently, Gosalawit-Utke and coworkers illustrated that confinement of MgH_2_, LiBH_4_ and NaAlH_4_ in carbon based scaffolds can facilitate improved hydrogen release, uptake kinetics, reversible hydrogen storage capacity and more favorable thermodynamic properties [220,221,222]. To increase their surface area, carbon aerogels were first activated by CO_2_ before the confinement of NaAlH_4_ [224]. It was found that the hydrogen desorption kinetics decreased with increasing surface area and the hydrogen storage capacity is more stable and decreases less during continuous hydrogen release and uptake cycles. In fact, the available amount of hydrogen (2.7 wt % H_2_) was more than doubled compared to the nanoconfinement in the non-activated carbon aerogel (1.3 wt % H_2_). Hydrogen desorption kinetics and reversible hydrogen storage properties of 0.55LiBH_4_-0.45Mg(BH_4_)_2_ melt-infiltrated nanoporous carbon aerogels were also studied [223]. Nanoconfinement of 0.55LiBH_4_-0.45Mg(BH_4_)_2_ in high surface area carbon aerogel appears to facilitate hydrogen release illustrated by release of 13.3 wt % H_2_ (93%) and only 8.4 wt % H_2_ (58%) from bulk hydride in the first cycle under the same physical condition. Notably, nanoconfinement also appear to have a beneficial effect on hydrogen uptake, since 8.3 wt % H_2_ (58%) is released from the high surface area scaffold and only 3.1 wt % H_2_ (22%) from the bulk sample during the fourth hydrogen release.

## 5. Conclusions and Outlook

### 5.1. Conclusions

Polymer aerogels and their derivative aerogels have been used to bridge the nanoscale porous structures of aerogel materials to practical macro-scale applications, due to their unique structures and properties such as 3D porous networks, high specific surface area, low density, low dielectric constant and high mechanical properties. In this review, the most recent progress in preparation, structures and properties of polymer- or carbon-based aerogels as well as their potential applications in various fields including energy storage, adsorption, thermal insulation and flame retardancy are introduced in details. Furthermore, with successful implementation, these highly porous structures of aerogels offer a larger range of applicability and, often, enhanced performance than their primitive organic precursors.

Regarding the preparation, generally, the most elementary step is the sol-gel process, *i.e.*, the transition of a system of colloidal particles in a solution into a disordered, branched, continuous network. Another important process is drying, which has been successfully developed, demonstrating the feasibility in preparing high quality polymer- and carbon-based aerogels. Among various drying methods, supercritical drying technique is the most effective method to obtain well-defined structures, while freeze-drying is a much easier, more economic and environmentally friendly way. Ambient pressure drying can be applied in large-scale industrial production, whereas it may result in environment pollution for the evaporation of solvents. For microwave drying and vacuum drying, they usually produce large pores or even induce the collapse of materials. Therefore, most of the drying methods reviewed in this paper require further improvement to produce aerogels with high porosity, low density and better mechanical properties through a low cost and environmentally friendly way. In order to achieve this goal, a better understanding of the preparation and drying mechanism of aerogels is necessary.

In the aspect of potential applications, the unique structures and properties of polymer/carbon aerogels and their hybrid aerogels make them promising candidates in many practical areas. Due to their lightweight, low thermal conductivity and low speed of sound, aerogels are hopeful thermal and acoustic insulation materials, which can be applied in building construction, vehicles, automobiles or space suit. The high surface area, high porosity and low density derived from the 3D porous network of aerogels ensure their practical applications in the fields of catalysis, sensors, adsorption and fuel storage. More importantly, unlike traditional silica aerogels, polymer-based aerogels with much enhanced mechanical properties can meet the requirements of industry and even severe demand in aerospace. Meanwhile, carbon aerogels are electrically conductive, which can be further applied in the fields of energy storage such as supercapacitors and lithium ion batteries. Finally, the feasibility of fabricating polymer-based hybrid aerogels by incorporating nanofillers can endow the aerogels with multi-functions. Therefore, polymer- or carbon-based aerogels and their derived hybrid aerogels with diversified structures and properties significantly extend the practical applications of aerogel-based materials.

### 5.2. Challenges and Outlook

Although new kinds of aerogel are continuously added to the aerogel family, the species of aerogels are still limited. Therefore, creating novel aerogels, either single-component or hybrid, is urgently needed. The studies on the single-component aerogel are fundamental, scientific and potentially applicable, while the studies on the hybrid aerogels are practical, technical and direct applicable. To create a single-component aerogel with the novel composition is relatively difficult but fundamentally valuable. A series of new aerogels (e.g., chalcogenide aerogel, CNT aerogel, graphene aerogel, diamond aerogel, silicon imidonitride aerogel, *etc.*) were successively invented in the past few decades. The next booming class may be carbide aerogel or single-element (mainly metal) aerogels. However, single-component aerogels are restricted in applications by their unicity. Hence, from a more practical view, hybrid aerogels with practical and smart functions should be designed and prepared. Combining polymer and carbon aerogels with nanofillers (e.g., graphene, carbon nanotube, nanofiber and nanoclays) is a promising strategy for producing aerogels with excellent properties and multi-functions.

Compared with the other applications, industrial applications can directly benefit human beings. However, due to the bad formability or mechanical properties, most of the aerogels are difficult to use in industry at the present stage. Besides, low yield and high cost also limit the commercialization of the aerogels. Thus, aerogel research is still developing and has a long way to go. The preparation of novel single-component aerogels, material design of hybrid aerogels and industrial application need to be further studied and investigated so as to give this state of matter a bright future.
